# Challenges in Reconstruction and Limb Lengthening Surgery for Congenital Lower Limb Deficiencies

**DOI:** 10.3390/jcm15186951

**Published:** 2026-09-08

**Authors:** Milud Shadi, Eliza Kortus, Piotr Janusz

**Affiliations:** Department of Spine Disorders and Pediatric Orthopedics, Wiktor Dega Clinical Hospital, Poznan University of Medical Sciences, 61-701 Poznań, Poland; eliza.kortus@gmail.com (E.K.); mdpjanusz@gmail.com (P.J.)

**Keywords:** congenital lower limb deficiency, preparatory reconstructive surgery, treatment strategy

## Abstract

**Background/Objectives**: Lower limb congenital deficiencies represent challenging disorders that require a complex strategy for limb reconstruction and lengthening procedures guided by the anatomical type and severity of the deformity. This review aims to summarize current treatment approaches in limb reconstruction and lengthening for patients with common congenital lower limb deficiencies, including fibular hemimelia, congenital femoral deficiency, and tibial hemimelia. **Methods**: A literature search was conducted using major databases, complemented by the senior author’s clinical experience and case series, to identify relevant studies and current procedures for the management of common congenital lower limb deficiencies, including limb reconstruction and lengthening techniques. **Results**: The various clinical presentation and classification systems of congenital lower limb deficiencies are presented, as well as up to date surgical techniques including limb reconstruction and lengthening. These approaches demonstrated effectiveness in congenital limb deficiencies management by facilitating joint stabilization and subsequent limb lengthening, leading to improved functional outcomes. However, significant challenges persist, including prolonged treatment duration and patient discomfort. Modern surgical approaches have significantly improved the outcomes of treatment for congenital limb deficiencies. However, the complexity of these clinical entities requires individualized treatment strategies. **Conclusions**: Successful management of congenital lower limb deficiencies relies on individualized, patient-specific treatment planning, incorporating preparatory surgical interventions and sequential limb lengthening procedures to maximize functional outcomes.

## 1. Introduction

Congenital lower limb deficiencies are among the most complex conditions encountered in paediatric orthopaedics. Fibular hemimelia is the most common form (1:40,000), followed by congenital femoral deficiency (1:50,000) and the much rarer tibial hemimelia (1:1,000,000) [1,2,3]. These conditions are frequently associated with marked limb-length discrepancies, multi-planar deformities, and significant functional impairment.

The management of these deformities is challenging because of the wide variability in presentation and the simultaneous involvement of multiple joints, bone segments, and soft-tissue structures. In addition to physical disability, these conditions can cause considerable functional limitations and cosmetic concerns. Compared with other reconstructive procedures, congenital limb deficiencies carry a significantly higher complication risk, emphasizing the necessity for staged, carefully sequenced treatment strategies [4].

The management of these deformities is challenging because of their wide variability in presentation and the simultaneous involvement of multiple joints, bone segments, and soft-tissue structures. Beyond physical disability, these conditions can cause substantial functional limitations, cosmetic concerns, social prejudice, and stigma. Compared with other reconstructive procedures, congenital limb deficiencies carry a significantly higher risk of complications, often requiring early, carefully sequenced, staged surgery throughout growth, with a potentially lasting psychological burden.

The main challenge in reconstruction is congenital ligamentous insufficiency, particularly around the knee and foot. Joint instability predisposes patients to recurrent subluxation or dislocation, and in severe cases, irreversible functional loss. Stabilizing these joints, especially the hip and knee, is frequently difficult, and in some cases impossible, because of soft-tissue imbalance, absent ligamentous structures, or abnormal joint morphology. These limitations highlight the importance of developing safe, methodical treatment strategies that prioritize joint protection [5,6].

**Figure 1:**
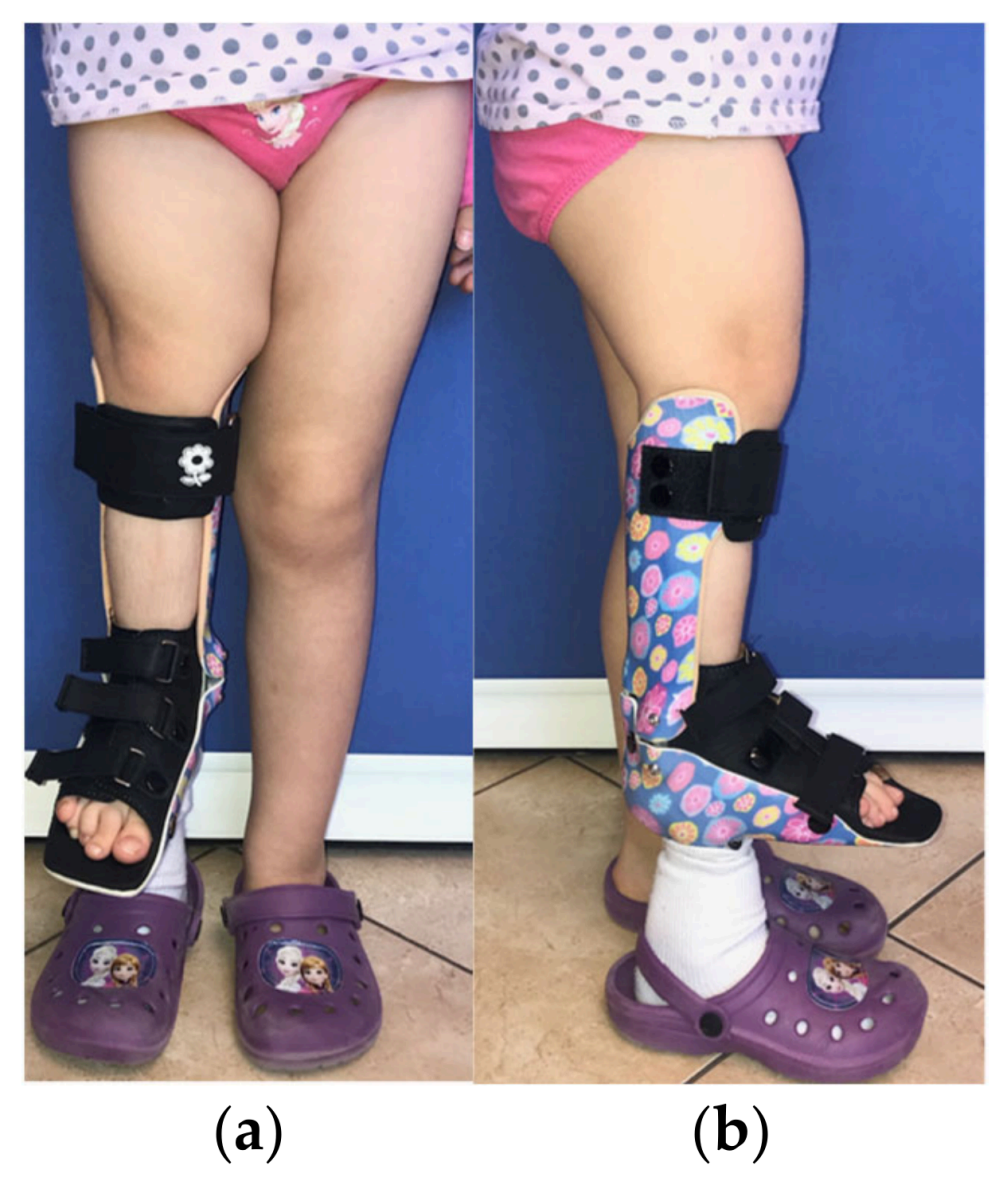
Severe shortening of the right lower limb with prosthetic fitting: (**a**) front view; (**b**) side view.

Conservative management is a cornerstone in the treatment of children with congenital limb deficiency and is commonly implemented before surgical intervention. Early strategies include patient-specific prosthetic fitting, structured prosthetic training, and targeted physiotherapy to support motor development, improve function, and maintain joint mobility. Psychological support, for both patients and families, plays an important role in promoting psychosocial adaptation. Together, these conservative measures aim to maximize functional independence, enhance quality of life, and prepare the child for potential future surgical procedures (Figure 1) [7,8,9].

Modern treatment concepts must account for the broad clinical variability and prioritize correct sequencing of interventions. Temporarily accepting limb shortening is often necessary to reduce soft-tissue tension and preserve joint integrity during reconstruction. Traditional approaches have often yielded suboptimal outcomes including hip subluxation or dislocation, recurrent deformities, and failure to achieve limb-length equalization at maturity [10].

A structured, prioritized approach stabilizing joints and restoring soft tissue balance before undertaking lengthening substantially improves long-term functional outcomes. Preparatory surgery is fundamental: unstable joints must be corrected and soft-tissue contractures released, even if this requires initial intentional shortening or staged reconstruction, as emphasized in Paley’s principles of reconstruction [11].

**Objective:** This article aims to explore current advances and challenges in limb lengthening and reconstructive surgery for congenital lower limb deficiencies, with separate consideration of CFD, fibular hemimelia, and tibial hemimelia, combining a critical review of the literature from the past 10 years with the authors’ personal experience.

## 2. Congenital Femoral Deficiency

Congenital femoral deficiency (CFD) is characterized by a wide spectrum of osseous and soft-tissue abnormalities affecting the hip, knee, ankle, and foot. The hip commonly demonstrates structural abnormalities, including mild to moderate acetabular dysplasia, femoral shortening with proximal deformities such as apparent coxa vara, anterior femoral bowing (“a bump”), retroversion, delayed ossification of the proximal femur, and in some cases femoral neck or subtrochanteric pseudarthrosis. Soft-tissue abnormalities are frequent and may include external rotation contractures (often resulting from a tight piriformis), hip flexion contractures involving the rectus femoris, iliopsoas, or tensor fascia lata, and abduction contractures due to tightness of the gluteus medius/minimus or the tensor fascia lata [11,12,13].

At the knee, patients may present with instability arising from anterior cruciate ligament (ACL) and/or posterior cruciate ligament (PCL) deficiency. Additional deformities may include distal femoral valgus related to lateral condylar hypoplasia, knee flexion contracture, hamstring shortening, and in some cases patellar subluxation or dislocation.

**Figure 2:**
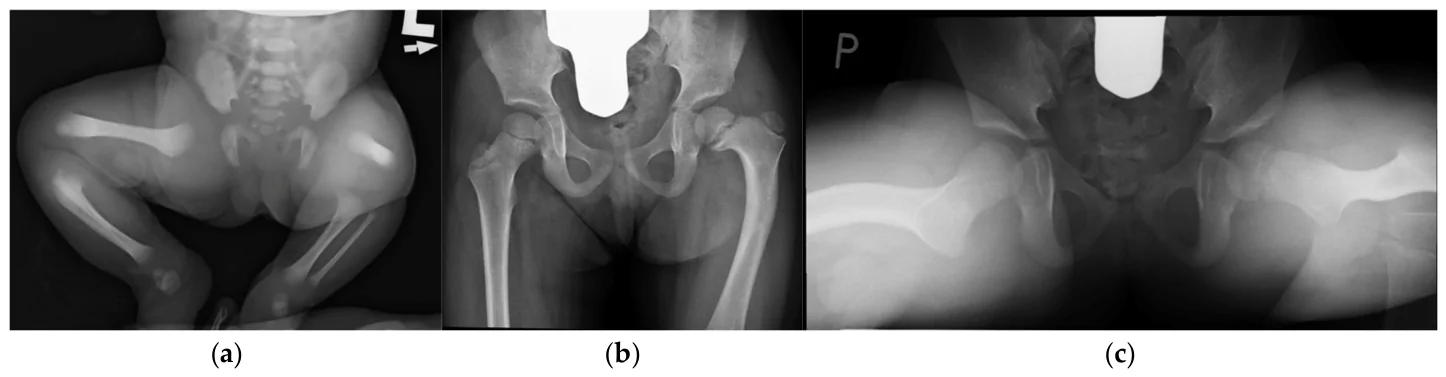
Radiographic views of the lower limb in a patient with right femoral shortening: (**a**) radiograph obtained just after birth; (**b**) pelvic radiograph at 3.5 years of age showing CFD type 1A3; (**c**) Rippstein position radiograph demonstrating proximal femoral retroversion. P—right.

At the foot and ankle, characteristic findings include equinovalgus deformity and the presence of a ball-and-socket ankle joint. CFD frequently occurs in association with ipsilateral fibular hemimelia (Figure 2) [12].

### 2.1. Clinical Examination

**Figure 3:**
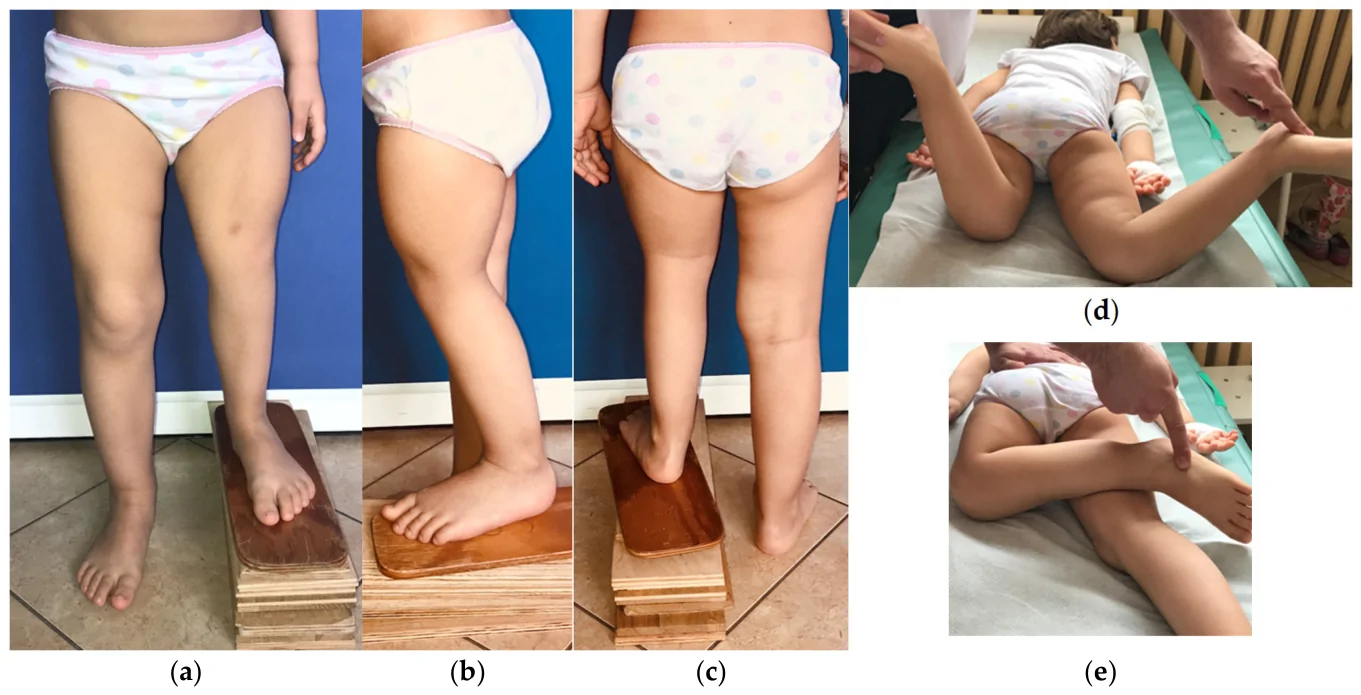
Clinical views of a 3.5-year-old patient with CFD and 8 cm shortening of the left lower limb: (**a**–**c**) general clinical views; (**d**) limitation of internal rotation; (**e**) excessive external rotation of the hip.

Clinical evaluation begins with a thorough assessment of the hip. Hip range of motion is examined in abduction, adduction, and flexion, followed by the Thomas test to identify fixed flexion deformity. Internal and external rotation are assessed to identify rotational abnormalities. Soft-tissue tightness is evaluated using the popliteal angle, Ely test, and Ober test to detect hamstring or iliotibial tract shortening. Knee examination includes careful assessment of flexion and extension. A knee flexion contracture of more than 10° is considered clinically significant and must be corrected before initiating limb lengthening. Anteroposterior knee stability is assessed with the Lachman test, whereas rotational instability, often caused by tight iliotibial tract or biceps femoris, is evaluated separately, as this may predispose to rotational subluxation of the knee and patella. Patellar stability is assessed using the “thumb push” test to identify any tendency toward lateral instability. The foot and ankle are examined last to identify associated abnormalities, particularly those linked to fibular hemimelia or other congenital malformations (Figure 3) [11,12,13].

### 2.2. Diagnostic Imaging

**Figure 4:**
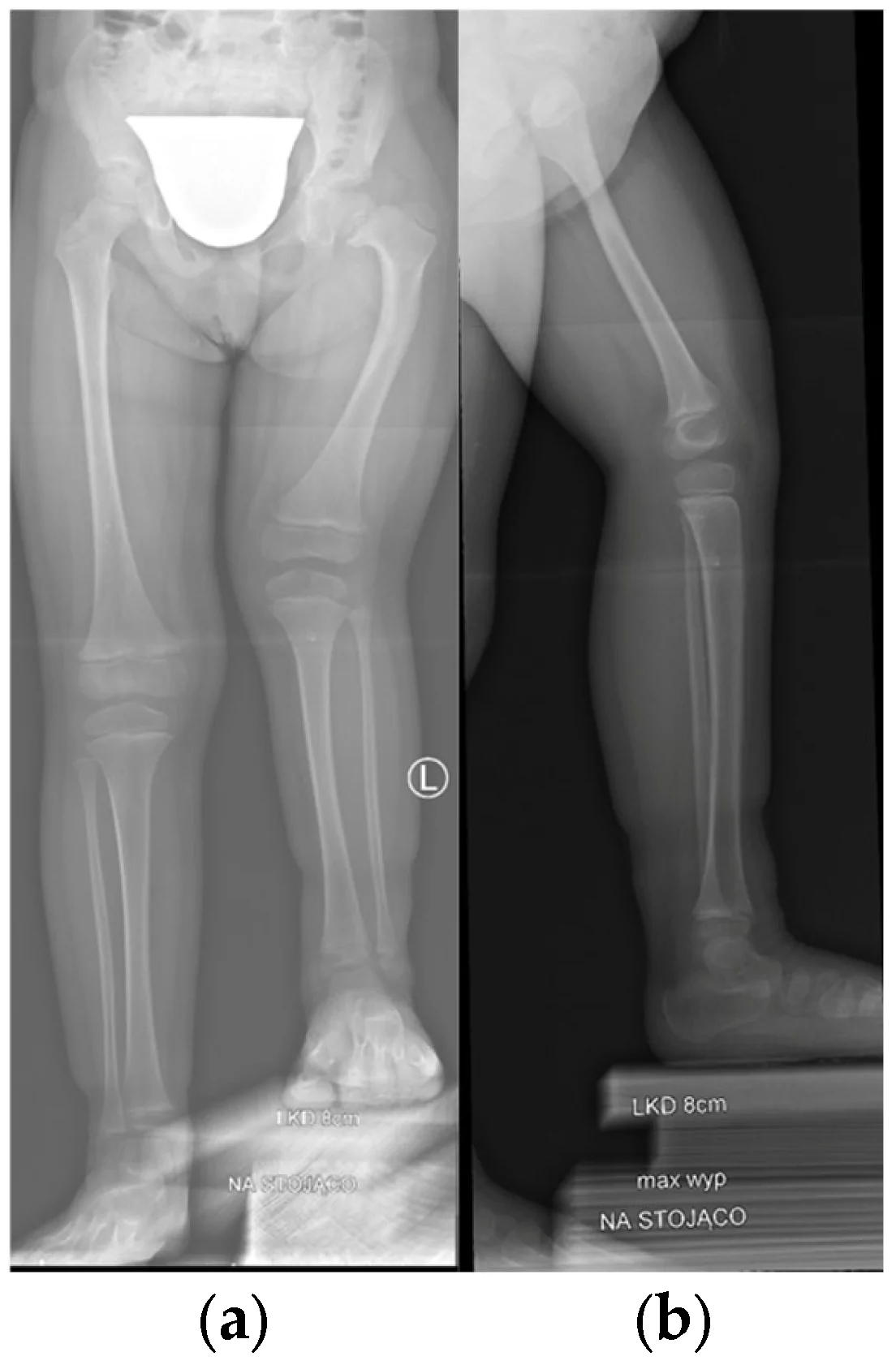
Standing radiographs of a 3.5-year-old patient with left-sided CFD type 1A3 and 9 cm shortening of the left lower limb: (**a**) anteroposterior; (**b**) lateral view. L—left, LKD—left lower limb, NA STOJĄCO—standing position, max wyp—complete knee extension.

Radiographic imaging plays a central role in diagnosing CFD and in planning surgical treatment. Standard radiographs include anteroposterior and lateral supine “pulled-down” views encompassing the pelvis and entire lower limbs. These allow evaluation of the acetabular index (AI), center-edge angle (CEA), CFD classification, femoral length, and limb-length discrepancy (LLD), including prediction of final LLD at skeletal maturity. Long lateral radiographs are essential for assessing knee flexion deformities (Figure 4).

**Figure 5:**
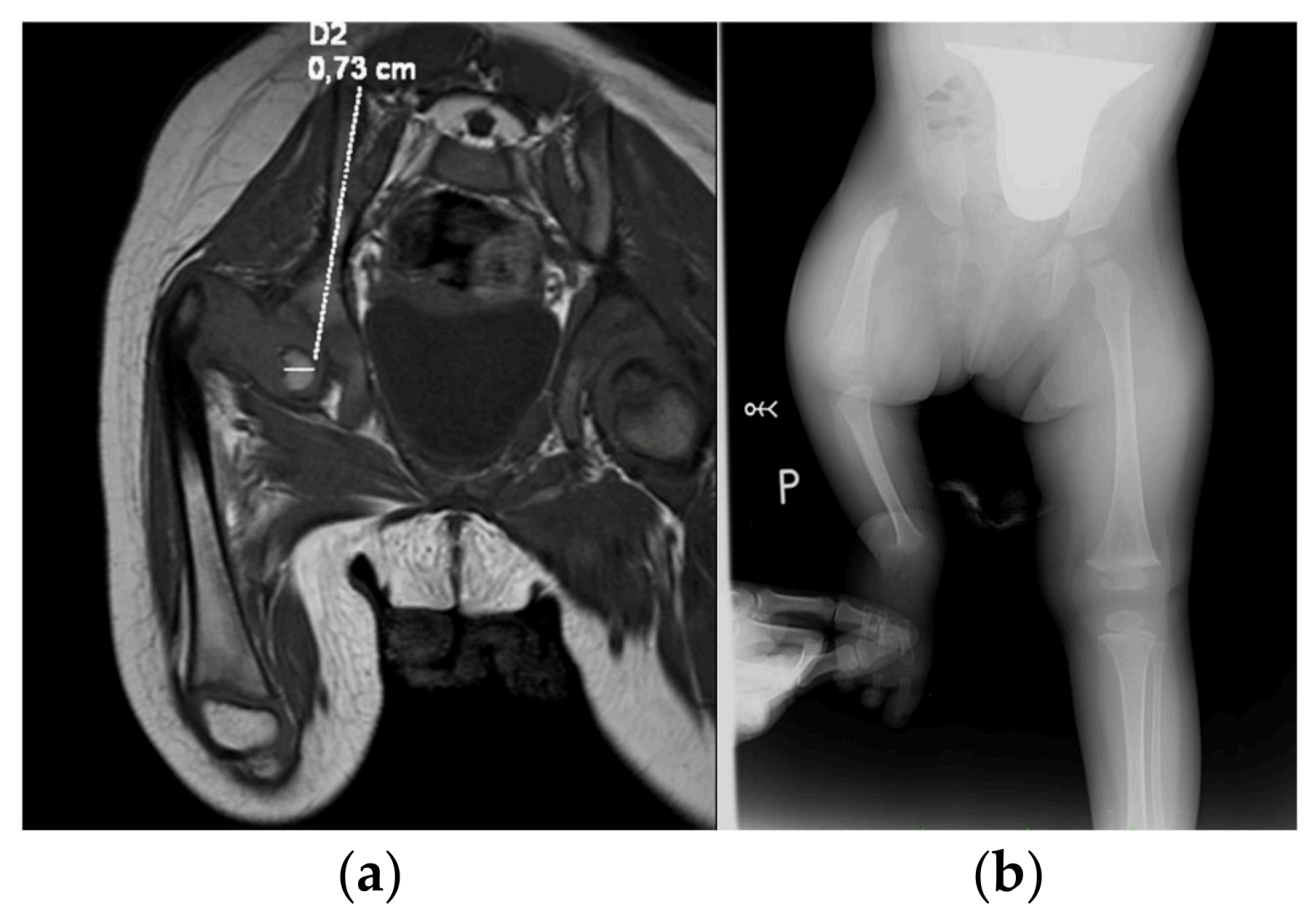
(**a**) MRI assessment of the hip demonstrating detailed visualization of the cartilaginous femoral head and assessment of hip stability compared with (**b**) plain radiography. D2—distance, P—right, figure o<-< means—supine position.

**Figure 6:**
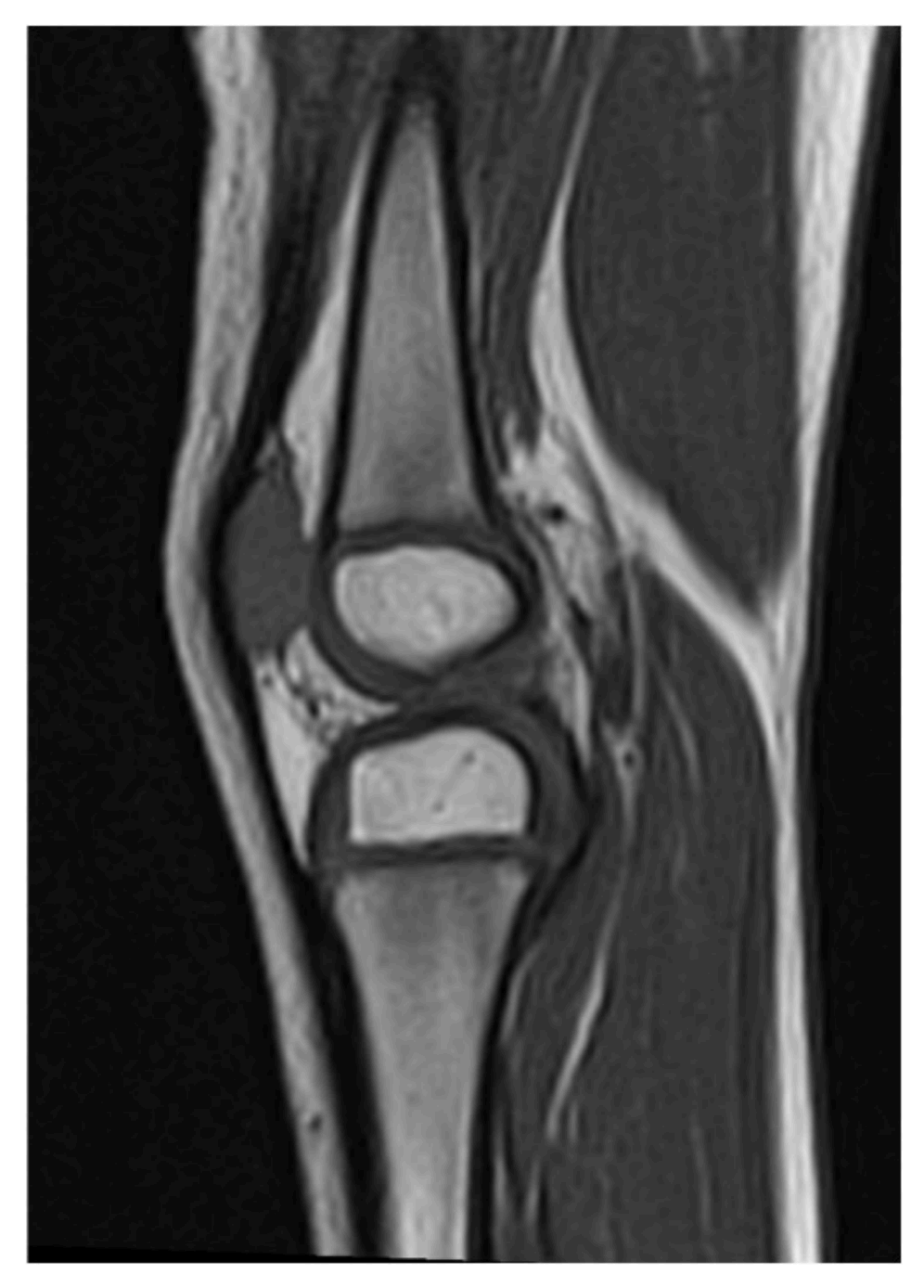
MRI of the left knee demonstrating ACL and PCL deficiency.

Magnetic resonance imaging (MRI) is particularly valuable in Paley types 1B and 2 after 18 months of age, as it enables visualization of the cartilaginous femoral head, assessment of hip stability, characterization of soft-tissue structures, and evaluation of continuity between the femoral head and shaft. MRI also demonstrates varying degree of abnormality in both bony and soft tissue, anatomy also provides important information regarding cruciate ligament integrity (Figure 5 and Figure 6) [11,12].

**Figure 7:**
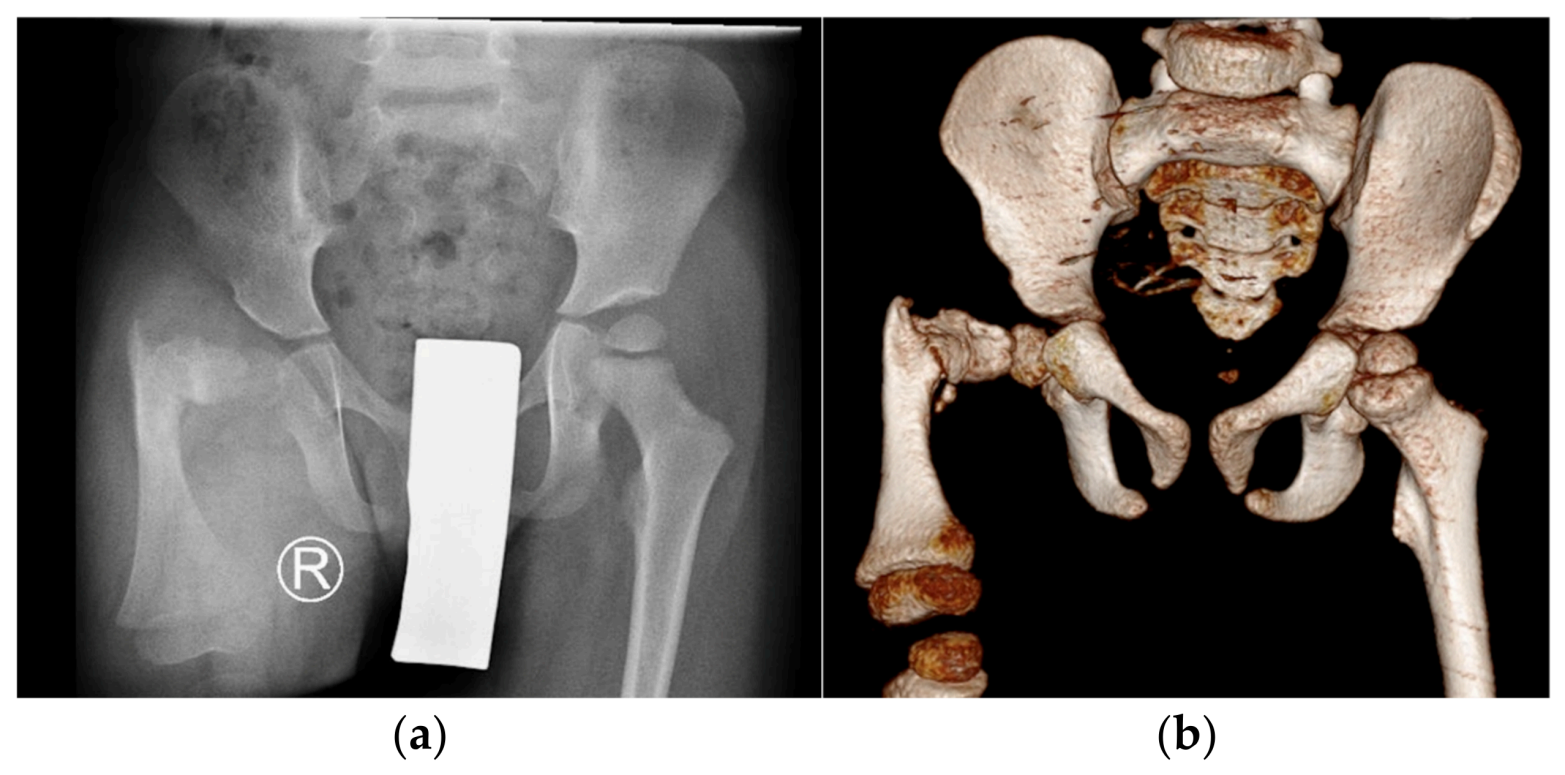
Imaging studies of a 5-year-old patient with right-sided CFD type 1B combined type (1B3): (**a**) radiograph; (**b**) 3D CT reconstruction. R—right.

Computed tomography (CT) with 3-dimensional reconstruction and 3D printing preoperatively is highly useful in defining complex bony deformities and supporting detailed preoperative planning, ultimately helping to optimize surgical outcomes (Figure 7) [14].

### 2.3. Classification Systems

Several classification systems have been proposed for congenital femoral deficiency (CFD), formerly known as proximal femoral focal deficiency (PFFD), with the Aitken, Pappas, and Paley classifications being the most commonly used [11,15,16].

The Aitken classification is one of the earliest and most widely recognized systems for congenital femoral deficiency. It is based primarily on radiographic assessment of the proximal femur and hip joint and categorizes patients according to the severity of femoral deficiency and acetabular development. Its primary advantage lies in its simplicity and ease of application in routine clinical practice. Furthermore, it enables standardized communication between orthopedic surgeons and radiologists. However, the Aitken classification has important limitations. It focuses mainly on proximal femoral anatomy and does not adequately address associated deformities involving the knee, ankle, or foot. In addition, it provides limited guidance regarding reconstructive options and long-term functional outcomes [15].

The Pappas classification was developed to provide a more comprehensive description of congenital femoral abnormalities. This system includes a broader spectrum of anatomical variations and offers a detailed characterization of deformities. The principal advantage of the Pappas classification is its anatomical completeness, allowing clinicians to describe rare and complex presentations. Nevertheless, its clinical use is limited because of its complexity and large number of categories. As a result, it is less practical for routine clinical decision-making and has not achieved widespread acceptance in daily orthopedic practice [16].

**Figure 8:**
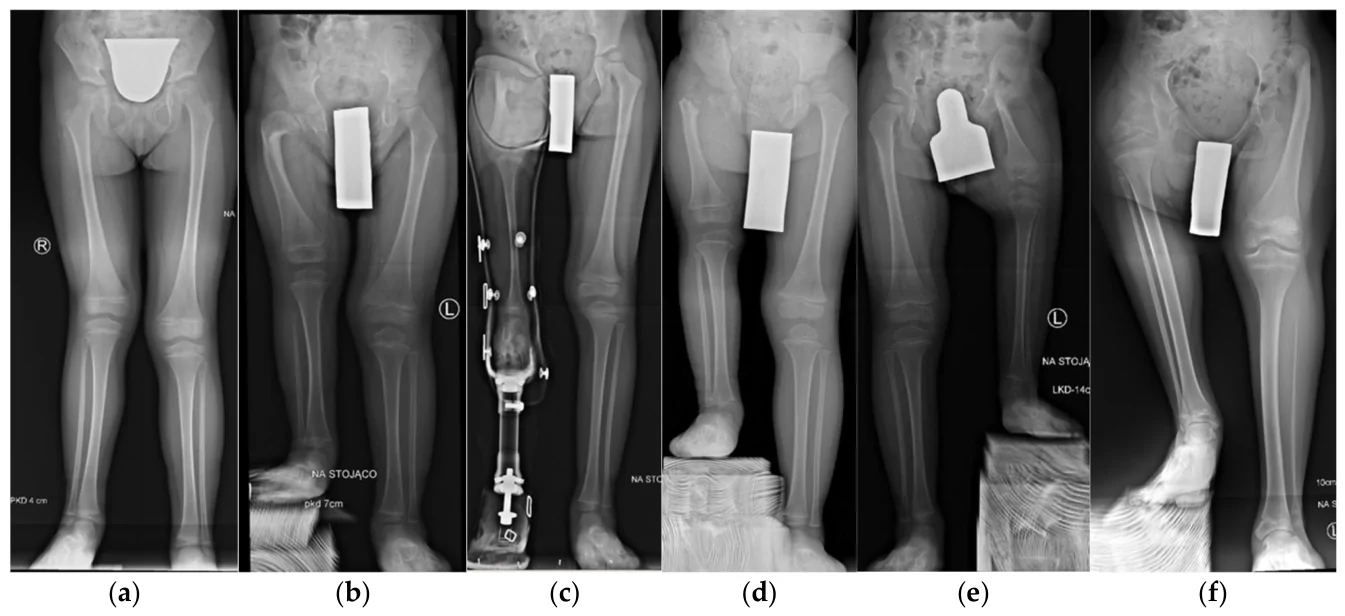
Common Paley types of CFD: (**a**) type 1A1 with normal ossification of the proximal femur; (**b**) type 1A3 with proximal femoral varus and retroversion; (**c**) type 1B3 demonstrating delayed ossification of the subtrochanteric region and femoral neck (combined type); (**d**) type 2A showing absence of the femoral neck with a mobile femoral head; (**e**) type 3A showing absence of the proximal femur with >45° of knee motion; (**f**) right femur type 3B, showing absence of the proximal femur with <45° of knee motion, and left hip type 2C. L—left, R—right, LKD—left lower limb, PKD—right lower limb, NA STOJĄCO—standing position.

To overcome these limitations, Paley introduced a treatment-oriented classification for congenital femoral deficiency. Unlike previous systems, the Paley classification not only describes anatomical abnormalities but also considers reconstructive potential and surgical management strategies. This approach allows direct correlation between classification type and treatment planning. The system also incorporates associated hip and knee pathologies, making it particularly useful for modern limb reconstruction procedures. Its primary drawback is the requirement for detailed clinical and radiological evaluation, sometimes including advanced imaging studies. Consequently, the Paley classification is considered the most useful system for contemporary reconstructive decision-making [11,13] (Figure 8).

Type 1. The hip joint and femur can be reconstructed, the femoral head is present and connected to the femoral shaft, although delayed ossification or deformity may exist. This type is subdivided into the following:

- 1A: Normal ossification of femoral neck and stable hip;
- 1B: Delayed ossification of the femoral neck, which may appear discontinuous on radiographs but remains connected by cartilage.

CFD type 1 is the most common form and affected patients are generally good candidates for reconstruction and limb lengthening.

Type 2. Mobile pseudarthrosis of the proximal femur where the femoral head is present but not stably connected to the femoral shaft. Subtypes include the following:

- 2A: Femoral head present and mobile within the acetabulum;
- 2B: Femoral head present but stiff or minimally mobile.

Reconstruction is possible but more complex and requires extensive preparatory surgery to stabilize the hip joint.

Type 3. Absent femoral head; the hip joint is non-reconstructable. This type is subdivided into the following:

- 3A: with knee motion greater than 45°;
- 3B: with knee motion less than 45°.

Limb lengthening in Type 3 is generally not possible, and treatment typically relies on prosthetic reconstruction.

Type 4. Distal Femoral Deficiency where the proximal femur and hip joint are relatively normal, but there is deficiency of the distal femur. Reconstructive treatment may be considered depending on knee stability and overall function.

### 2.4. Treatment Strategy

Because CFD presents with a broad range of deformities, treatment must begin with restoration of joint stability and correction of soft-tissue imbalance before lengthening is considered. Preparatory surgery is essential to prevent severe complications such as posterior hip subluxation or dislocation, which represent major risks during lengthening.

Treatment planning is based on Paley classification type, reconstructability of the hip and knee, and the predicted final limb-length discrepancy using the multiplier method.

Prior to any lengthening procedure, both the hip and knee must be stable and properly aligned, and the femoral neck must be fully ossified. Preparatory surgeries are ideally performed between 2 and 3 years of age when the proximal femur completely ossified [11,13].

#### 2.4.1. SUPERhip and SUPERknee Procedures

The Systematic Utilitarian Procedure for Extremity Reconstruction (SUPERhip and SUPERknee) is designed to correct all hip and knee deformities comprehensively during a single operative session, restoring both anatomical alignment and biomechanical function [11,13,17,18].

##### SUPERhip Procedure

**Figure 9:**
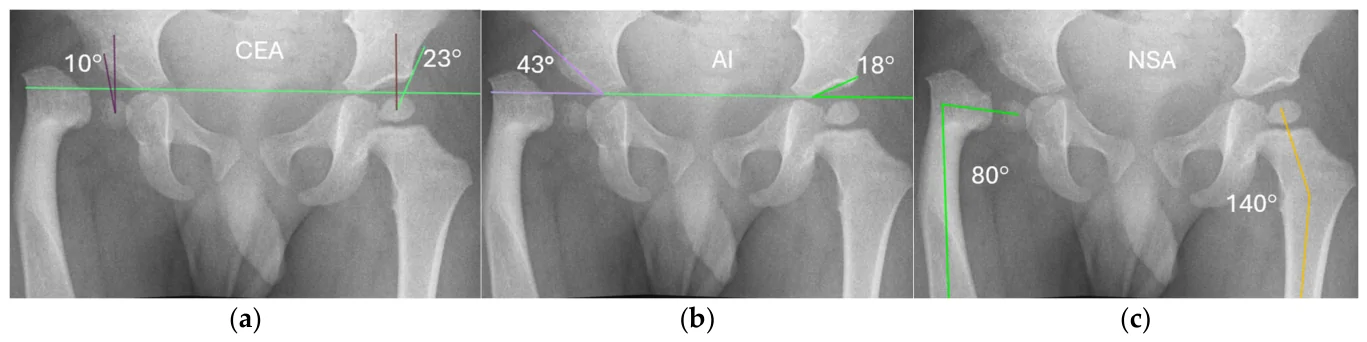
Radiographic hip measurements: (**a**) center-edge angle (CEA), normal ≥ 20°; (**b**) acetabular index angle (AI), normal ≤ 30°; (**c**) neck-shaft angle (NSA), normal ~ 130°.

**Figure 10:**
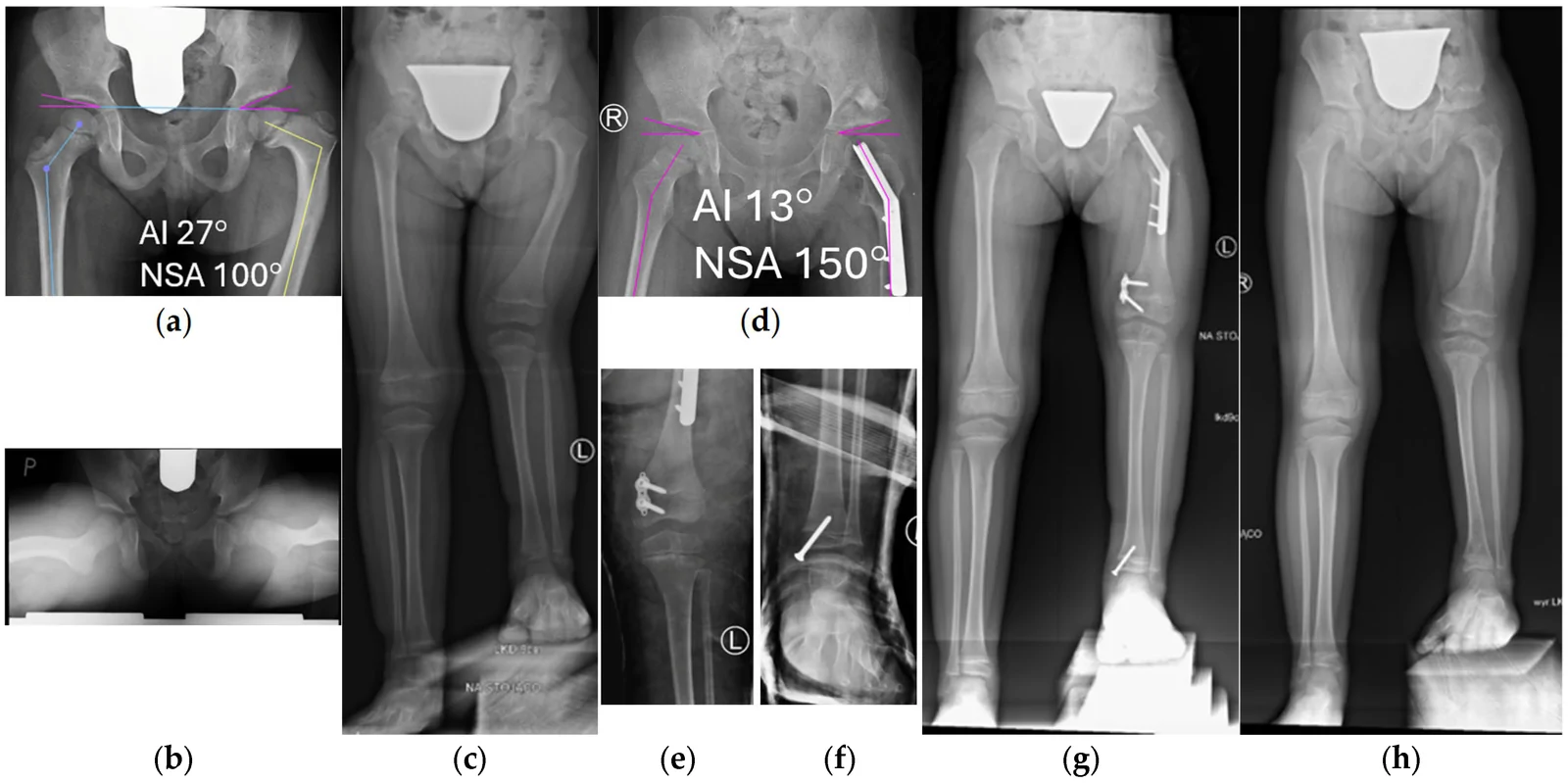
Radiographs demonstrating treatment of left CFD type 1A3: (**a**) anteroposterior view; (**b**) reduced femoral anteversion angle; (**c**) standing radiograph showing knee valgus, proximal migration of the lateral malleolus and 9 cm LLD; (**d**) pelvic radiograph after SUPERhip procedure; (**e**) 8-plate fixation for correction of knee valgus; (**f**) medial malleolar hemiepiphysiodesis performed to prevent ankle valgus; (**g**) standing radiograph obtained 1 year later showing restoration of the femoral axis and reconstructed hip prior to hardware removal; (**h**) standing radiograph after hardware removal. LKD—left lower limb, NA STOJĄCO—standing position, L—left, R—right, AI—acetabular index, NSA—neck-shaft angle.

Fundamental components of SUPERhip procedure include soft-tissue releases addressing hip flexion and abduction contractures, psoas recession, rectus femoris release, sciatic and femoral nerve decompression, fascia lata lengthening or resection, and abductor sliding when necessary. Procedure may include proximal femoral osteotomy to correct retroversion, varus, and anterior bowing, as well as pelvic osteotomy (Dega or triple osteotomy) when the center-edge angle (CEA) is ≤20° or the acetabular index (AI) is ≥30° [19]. Acetabular deficiency is usually lateral, posterior, or global. SUPERhip procedure restores proper hip containment and stability (Figure 9 and Figure 10) [17,18].

##### SUPERknee Procedure

**Figure 11:**
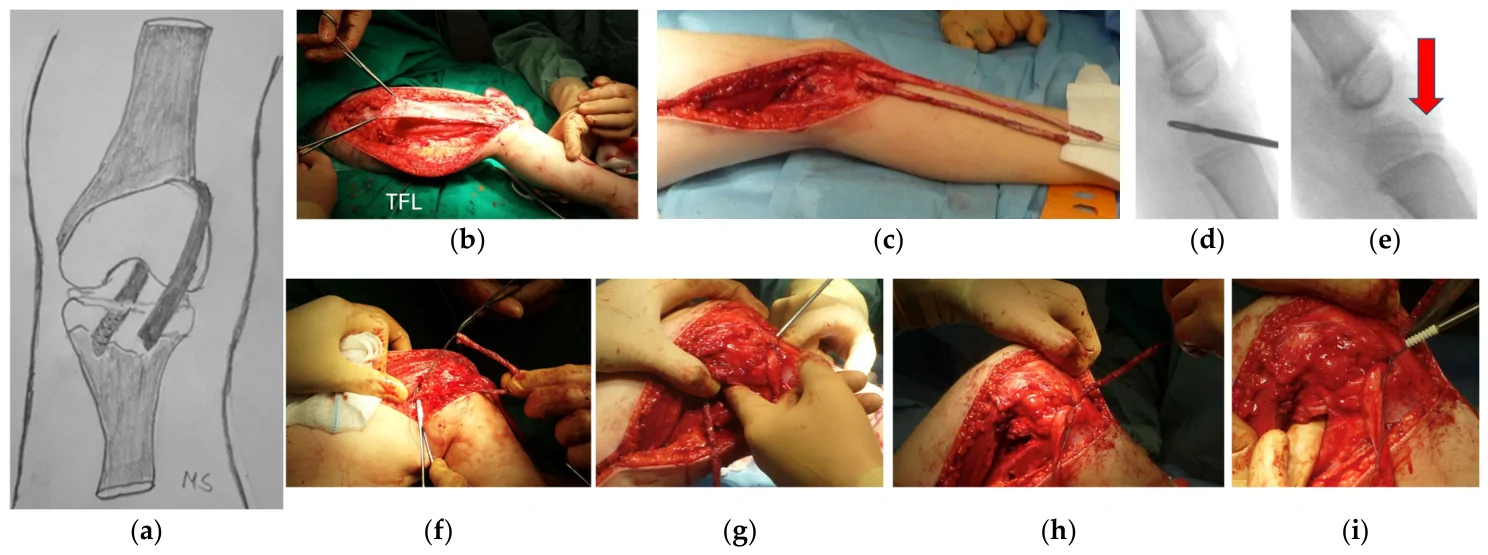
SUPERknee procedure using the reverse MacIntosh technique, an extra-articular reconstruction method utilizing the fascia lata: (**a**) schematic drawing of the final result ACL reconstruction; (**b**) preparation of the fascia lata; (**c**) preparation of the fascia lata graft to reconstruct the ACL and PCL ligaments; (**d**) intraoperative radiographic view showing preparation of the bone canal for graft passage; (**e**) red arrow indicating the canal and interference screw visible on the radiograph; (**f**,**g**) sequential steps of graft passage through the canal; (**h**) tightening of the ACL graft with knee flexion 90 degree; (**i**) insertion of the interference screw securing the graft.

SUPERknee procedure is indicated for patients with knee instability or absence of the cruciate ligaments [6]. The ACL is reconstructed using the MacIntosh technique, providing both intra- and extra-articular stabilization. The PCL is reconstructed using the reverse MacIntosh technique, an extra-articular method utilizing the fascia lata. This procedure is done simultaneously with SUPERhip to take advantage of the transected fascia lata. If the patella is subluxated or dislocated, the extensor mechanism can be reconstructed during the same surgery with Langenskiöld and Grammont procedures. If the knee is stable, resection of the fascia lata may be indicated to reduce soft-tissue tension during limb lengthening (Figure 11).

#### 2.4.2. Limb Lengthening in CFD

**Figure 12:**
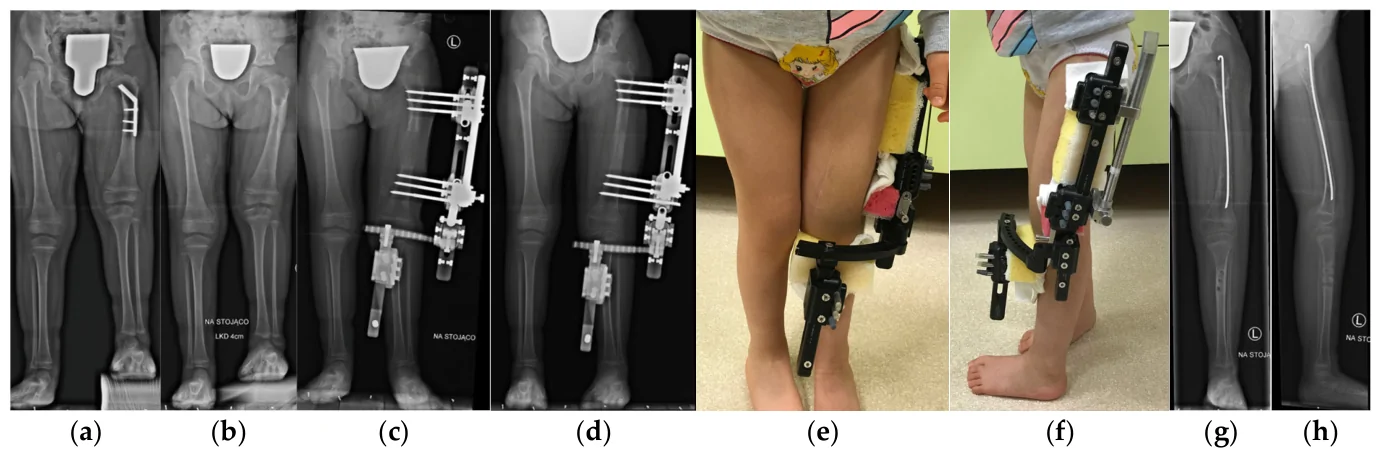
Radiographic and clinical views of left CFD type 1A: (**a**) standing radiograph after SUPERhip and SUPERknee procedures; (**b**) 5 cm shortening of the left femur before lengthening; (**c**) femoral lengthening using an MRS external fixator with a knee hinge; (**d**) consolidation phase after 5 cm femoral lengthening; (**e**,**f**) clinical views of the MRS external fixator; (**g**,**h**) prophylactic fixation with a Rush pin after MRS removal. L—left, LKD—left lower limb, NA STOJĄCO—standing position.

For young children (before age 4), the need for preparatory surgery depends on the severity of LLD. When preparatory surgery is required, limb lengthening typically begins one year after hip and knee reconstruction, usually at 3–4 years of age, with 5–8 cm achievable per lengthening cycle [5,20,21]. Lengthening is commonly performed using a distal femoral metaphyseal osteotomy and external fixation, often with knee-spanning hinges (e.g., hybrid or monolateral rail fixators-MRS). These devices protect the knee from subluxation during distraction (Figure 12) [21].

### 2.5. Postoperative Management

**Figure 13:**
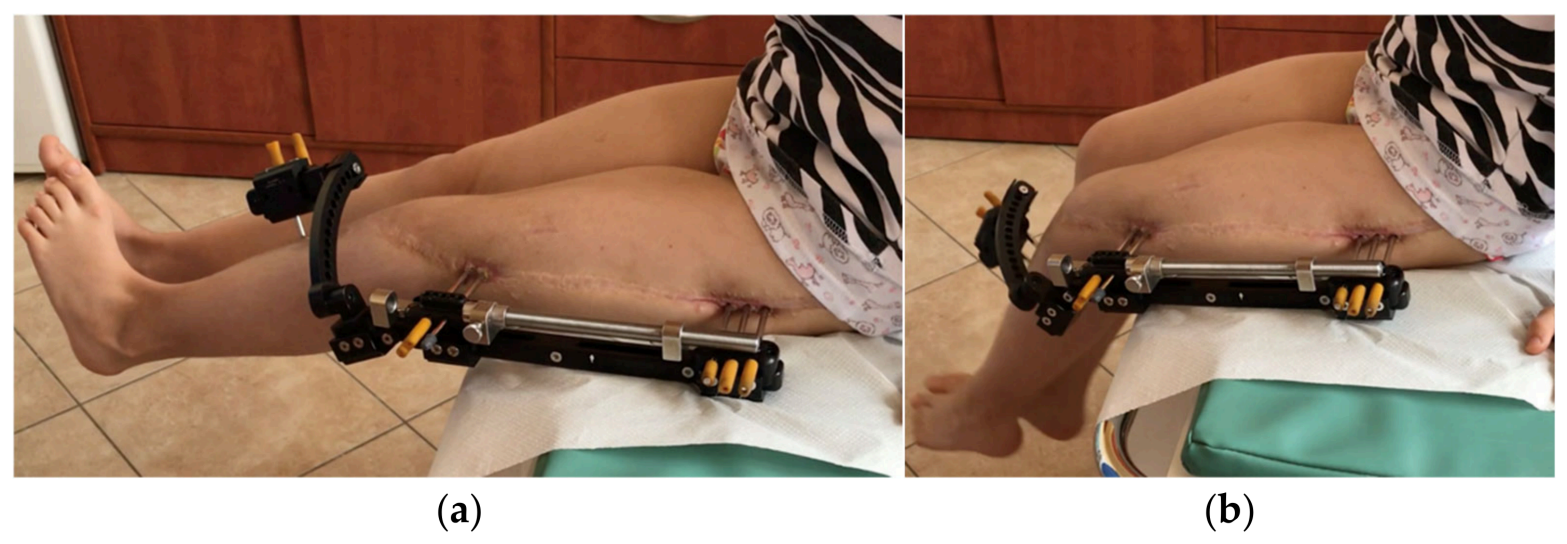
Early physiotherapy with unlocked knee hinge: (**a**) active knee extension; (**b**) active knee flexion.

Early physiotherapy begins on postoperative day one, with full weight-bearing as tolerated, and distraction starting on day five at a rate of 1 mm/day. Follow-up appointments are performed every two weeks for clinical and radiographic assessment, with ultrasonographic monitoring of bone regenerate formation and adjustments of the distraction rate based on regenerate quality (Figure 13).

The most common complication is pin-site infection, typically managed conservatively. Knee flexion limitation may occur and requires intensive physiotherapy. To prevent hip subluxation, the total lengthening should not exceed 25% of the femoral length.

After consolidation, the external fixator is removed and a prophylactic intramedullary rod is inserted to minimize the risk of fracture. The rods are usually removed one year later.

## 3. Fibular Hemimelia (FH)

Fibular hemimelia (FH) is the most common congenital cause of limb-length discrepancy. It presents with a broad spectrum of severity, ranging from mild fibular hypoplasia to complete fibular aplasia. Importantly, FH is not an isolated malformation of the fibula; rather, it represents a complex congenital disorder involving the entire lower limb. The principal problems include marked malalignment of the foot, with the longitudinal foot axis internally rotated by approximately 45° relative to the trans-malleolar axis. This severe torsional deformity results in a laterally positioned heel and medially oriented foot, typically with the foot deviated outward and downward, producing the clinical appearance of a fixed equinovalgus deformity. In reality, ankle motion occurs along an oblique rather than sagittal axis, which may lead to misinterpretation of this abnormal motion axis as a rigid equinus–valgus deformity. Additional common features include cruciate ligament deficiency with secondary knee deformity and instability, as well as a constant and often substantial limb-length discrepancy [22,23,24,25].

### 3.1. Clinical Examination

**Figure 14:**
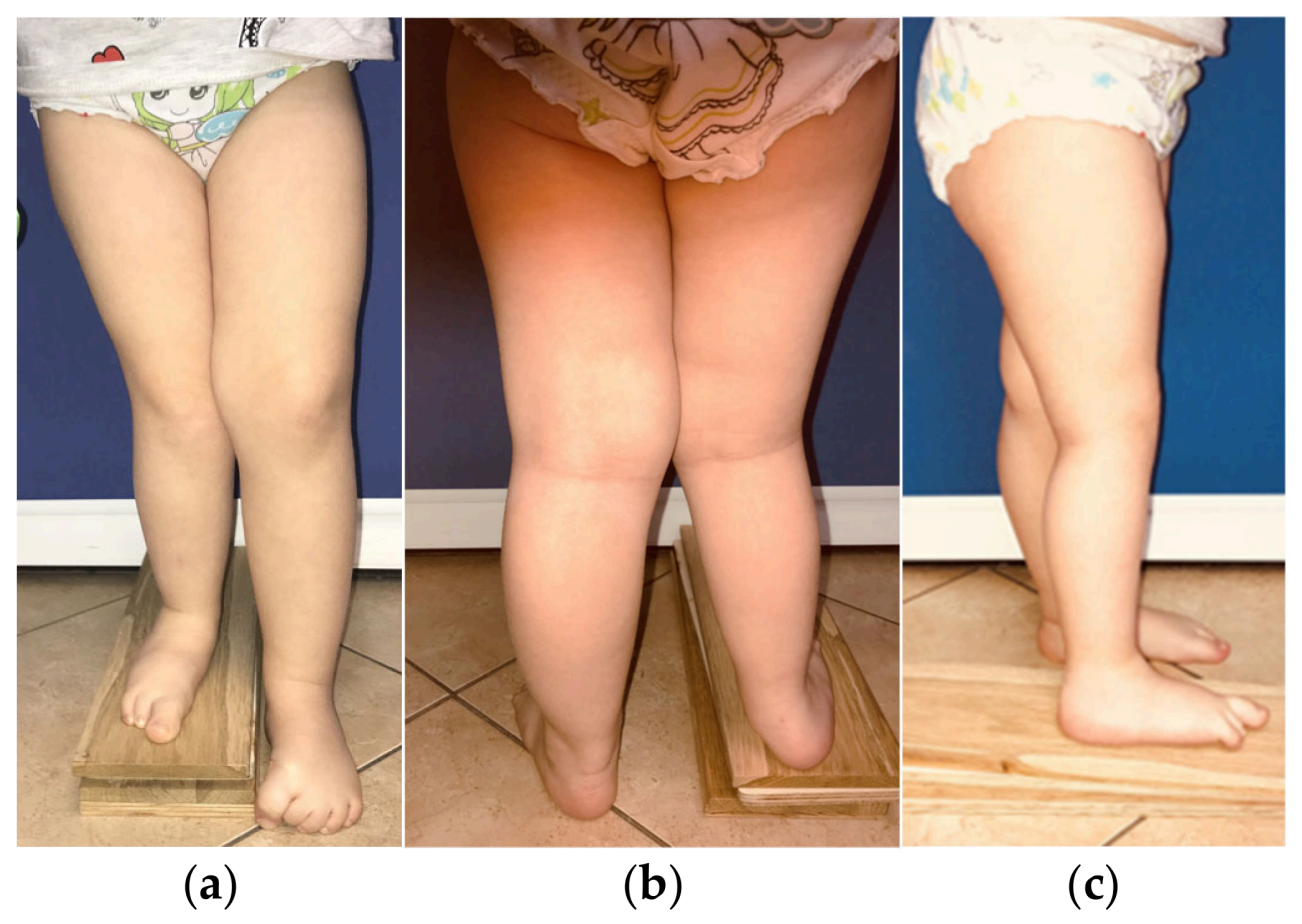
(**a**–**c**) Clinical views of right fibular hemimelia demonstrating limb shortening, valgus knee alignment, equinovalgus foot deformity, and deficiency of the lateral rays.

Evaluation must encompass the entire limb, as FH frequently coexists with other congenital abnormalities. Common associated findings include: hypoplasia of the lateral femoral condyle with resultant knee valgus, knee instability, patellar hypoplasia or instability, anteromedial tibial bowing with a characteristic skin dimple, ball-and-socket ankle configuration, ankle instability and absence of lateral foot rays, tarsal coalitions, particularly talocalcaneal fusions, significant foot deformities and, most commonly, equinovalgus and leg-length inequality (often severe). FH may also coexist with ipsilateral congenital femoral deficiency and with upper-limb anomalies such as those seen in FATCO syndrome (fibular aplasia, tibial campomelia, and oligosyndactyly) (Figure 14) [26].

### 3.2. Diagnostic Imaging

**Figure 15:**
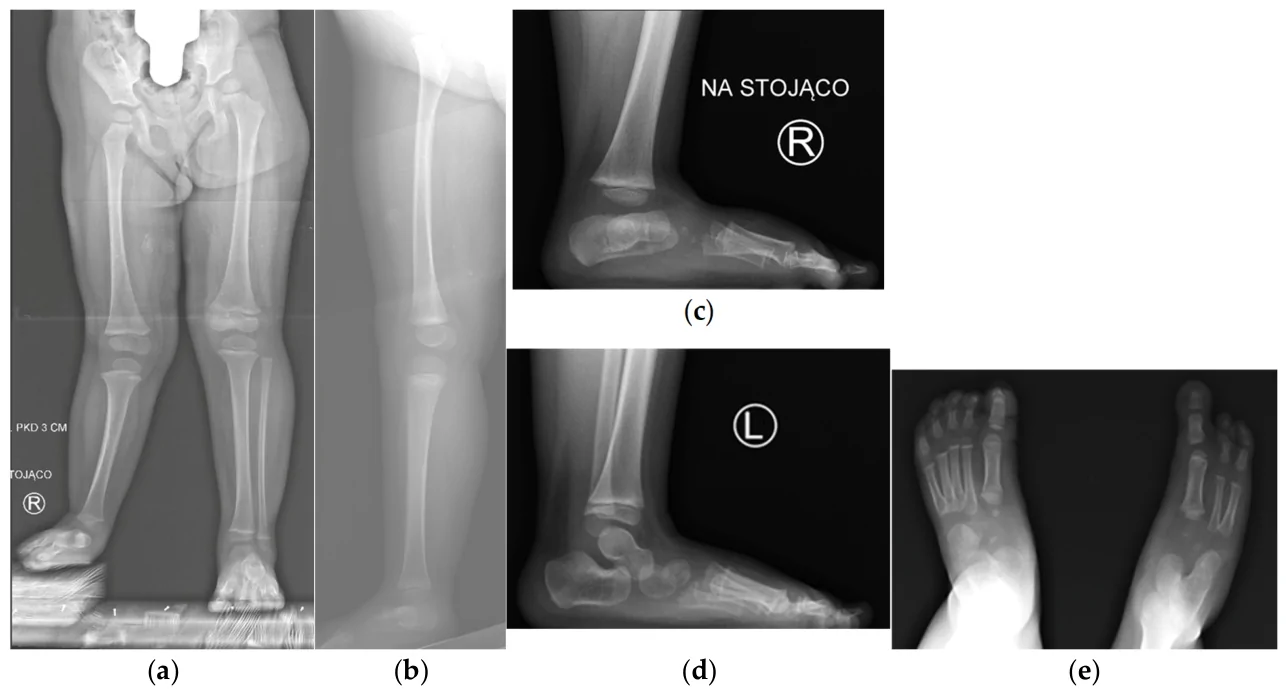
Radiographic views in fibular hemimelia: (**a**) full-length standing anteroposterior radiograph showing tibial shortening, valgus mechanical axis, ankle valgus; (**b**) lateral standing radiograph of the right limb with normal knee orientation; (**c**) lateral standing radiograph of the right foot demonstrating valgus alignment between the calcaneus and talus and talocalcaneal coalition; (**d**) lateral standing radiograph of a right foot (normal); (**e**) standing weight-bearing anteroposterior foot radiograph showing talocalcaneal coalition with valgus deformity and absence/hypoplasia of the lateral rays of the right foot. L—left, R—right, PKD—right lower limb, NA STOJĄCO—standing position.

Radiographic assessment in fibular hemimelia (FH) follows the same principles described for congenital femoral deficiency (CFD), including standard radiographic projections and advanced imaging when indicated. In ambulatory children, full-length standing anteroposterior radiographs are particularly important for assessing limb alignment, femoral and tibial deformities, and knee valgus. Lateral leg radiographs help evaluate tibial procurvatum. Dedicated foot and ankle radiographs, including anteroposterior, lateral, mortise, and Saltzman hindfoot alignment views, provide detailed assessment of foot deformities, ray deficiencies, ankle morphology, and hindfoot alignment (Figure 15) [22].

### 3.3. Classification Systems

Several classification systems have been proposed for fibular hemimelia. The Achterman–Kalamchi classification is based on the degree of fibular absence and remains widely used because of its simplicity and reproducibility. However, it does not adequately address associated foot, ankle, and knee abnormalities or treatment options [24].

Catagni’s classification is reconstruction-oriented and was developed based on Ilizarov limb reconstruction principles. It emphasizes deformity severity, foot and ankle abnormalities, and limb-salvage potential. While it is valuable for planning complex reconstructive procedures, it is more dependent on surgical expertise, less widely validated, and has not achieved broad international acceptance [27].

The Stanitski classification incorporates both fibular deficiency and foot deformity, providing a more comprehensive assessment of overall limb involvement. Nevertheless, its role in treatment planning remains limited [28].

The Birch classification focuses on foot function and predicted limb-length discrepancy, making it particularly useful when deciding between reconstruction and amputation. However, it provides less anatomical detail than other systems [29].

Nevertheless, the Paley classification has gained the widest acceptance among limb reconstruction surgeons because it integrates anatomical abnormalities, foot and ankle pathology, reconstructive potential, and surgical strategy, thereby providing the most comprehensive framework for contemporary management [22].

**Figure 16:**
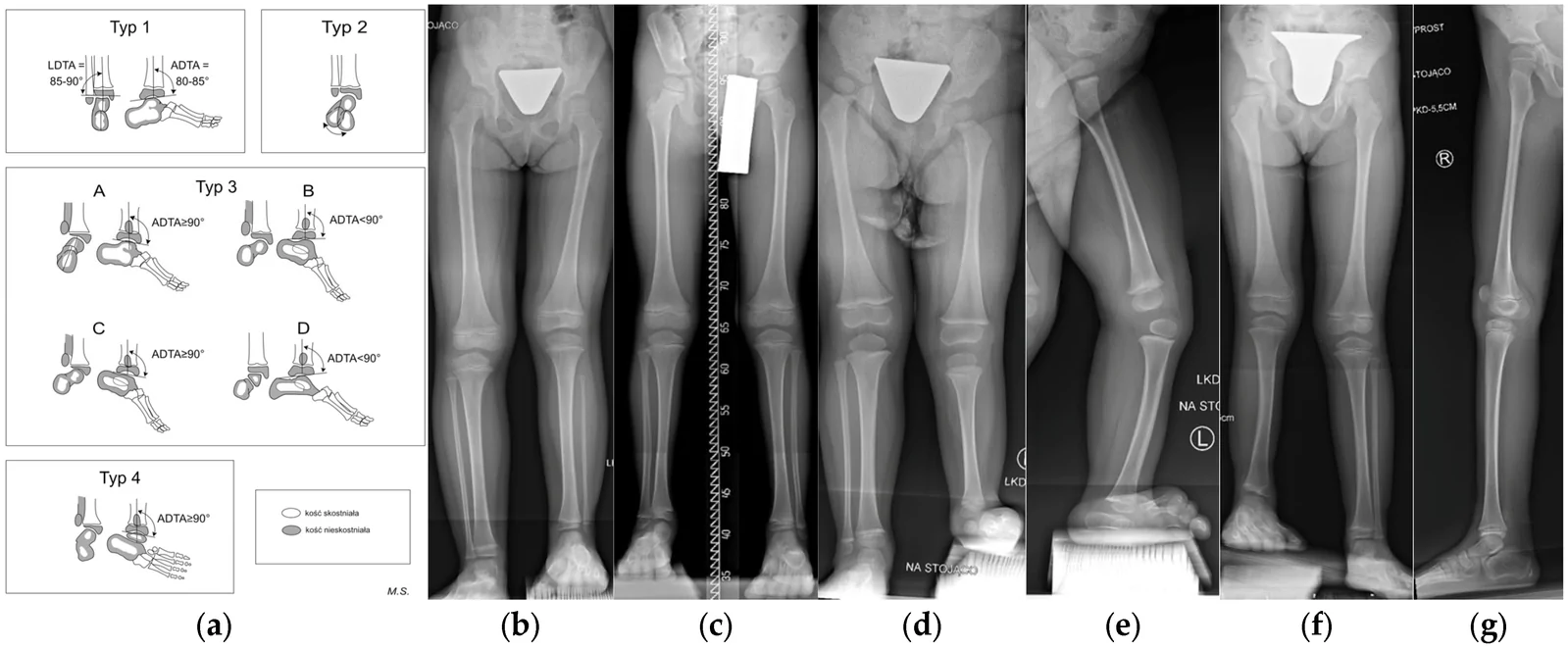
Illustration of the Paley classification of fibular hemimelia (FH): (**a**) type 1; (**b**) type 2; (**c**–**e**) type 3C; (**f**,**g**) type 4. L—left, R—right, LKD—left lower limb, NA STOJĄCO—standing position.

The Paley Classification (Figure 16) describes the ankle and subtalar joint deformities in detail and links each type to a specific reconstructive strategy [22,30]. It includes the following:

- Type 1: Stable, normal ankle;
- Type 2: Dynamic valgus ankle;
- Type 3: Fixed equinovalgus ankle, subdivided into: 3A (ankle type)*:* ankle maloriented in procurvatum and valgus; 3B (subtalar type): Subtalar coalition malunited in equinovalgus with lateral translation; 3C (combined type): Combined ankle and subtalar deformities; and 3D (talar type): Malorientation arising from subtalar joint abnormalities;
- Type 4: Fixed equinovarus ankle (clubfoot-type deformity).

### 3.4. Treatment Strategy

The overarching goal in treating FH is to restore a plantigrade, functional foot, achieve proper limb alignment, and correct limb-length discrepancy [22]. While achieving bone length is relatively straightforward with modern lengthening techniques, correcting the fixed equinovalgus deformity of the foot and ankle remains the greatest challenge [31,32]. Residual or recurrent deformity, especially ankle instability or fixed equinovalgus, remains the most frequent cause of unsatisfactory outcomes.

Two main treatment pathways exist: limb reconstruction or ablative procedures (e.g., Syme or Boyd amputation). Although amputation can produce reliable functional results [33,34], many families prefer limb reconstruction when feasible.

#### Principles of Reconstruction

Reconstructive treatment must address foot deformity, ankle and subtalar instability, soft-tissue contractures, limb axis malalignment, and limb-length discrepancy.

Importantly, foot correction must precede limb lengthening. Attempting lengthening without a stable, plantigrade foot substantially increases the risk of recurrence and treatment failure [22,30].

FH is particularly challenging because of maloriented ankle and subtalar joints, tarsal coalitions, tight fibular anlage causing lateral tethering, tight Achilles tendon, absence of lateral ankle support (fibular malleolus), and wedge-shaped distal tibial epiphysis contributing to lateral talar shift. Even with modern techniques, recurrent deformity in severe FH remains an expected risk [31,35].

### 3.5. Treatment Planning

The reconstructive life plan includes determining FH type using the Paley classification, selecting procedures aimed at achieving a plantigrade foot, correcting limb alignment, and predicting LLD and planning the number and timing of lengthening’s [22,30].

The reconstructive life plan is based on the Paley classification and aims to achieve a plantigrade foot, restore limb alignment, predict limb-length discrepancy, and plan the number and timing of lengthening procedures. Type 1 cases, with a stable, well-aligned and essentially normal ankle and mild shortening (DSM ≤ 5 cm), require no ankle reconstruction and can be managed with limb lengthening and/or contralateral epiphysiodesis. Type 2 cases with dynamic ankle valgus and preserved joint congruency are treated with the Shortening Osteotomy Realignment Distal Tibia (SHORDT), followed by limb lengthening. Severe cases with a stiff equinovalgus deformity require more complex reconstructive procedures, such as the SUPERankle procedure [22,36].

#### 3.5.1. SUPERankle Procedure

The SUPERankle procedure addresses complex deformities through a combination of bone and soft-tissue corrections. Depending on deformity severity, surgery may include the following:

Soft-tissue release, excision of the fibular anlage, osteotomy of talocalcaneal coalitions, and supramalleollar varus–dorsiflexion osteotomy.

**Figure 17:**
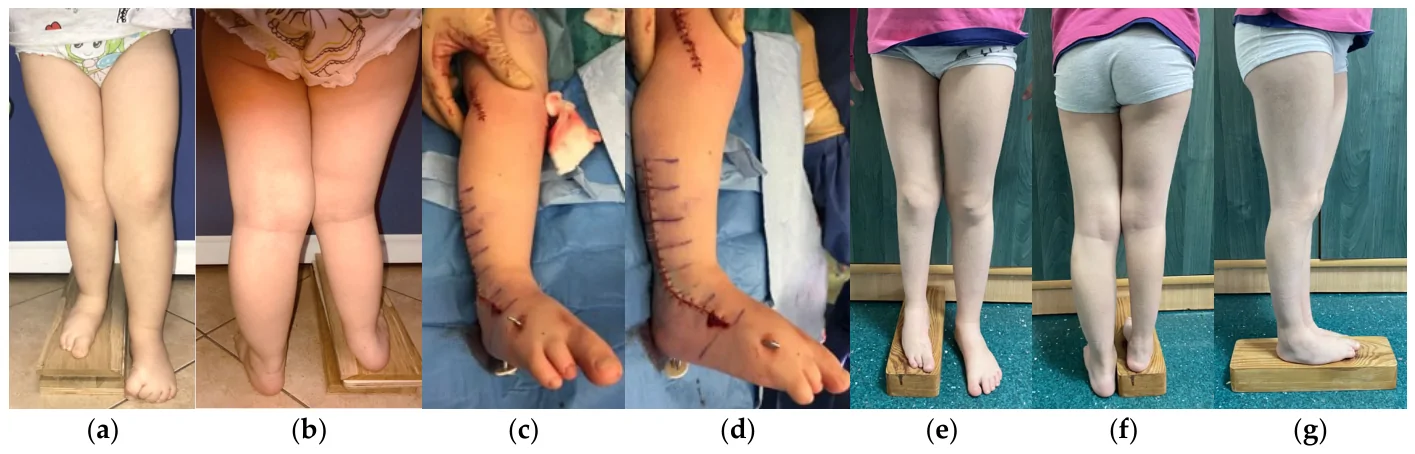
Clinical view of type 3C FH: (**a**,**b**) preoperative; (**c**,**d**) immediate postoperative appearance after SUPERankle procedure; (**e**–**g**) clinical result 1 year postoperatively.

**Figure 18:**
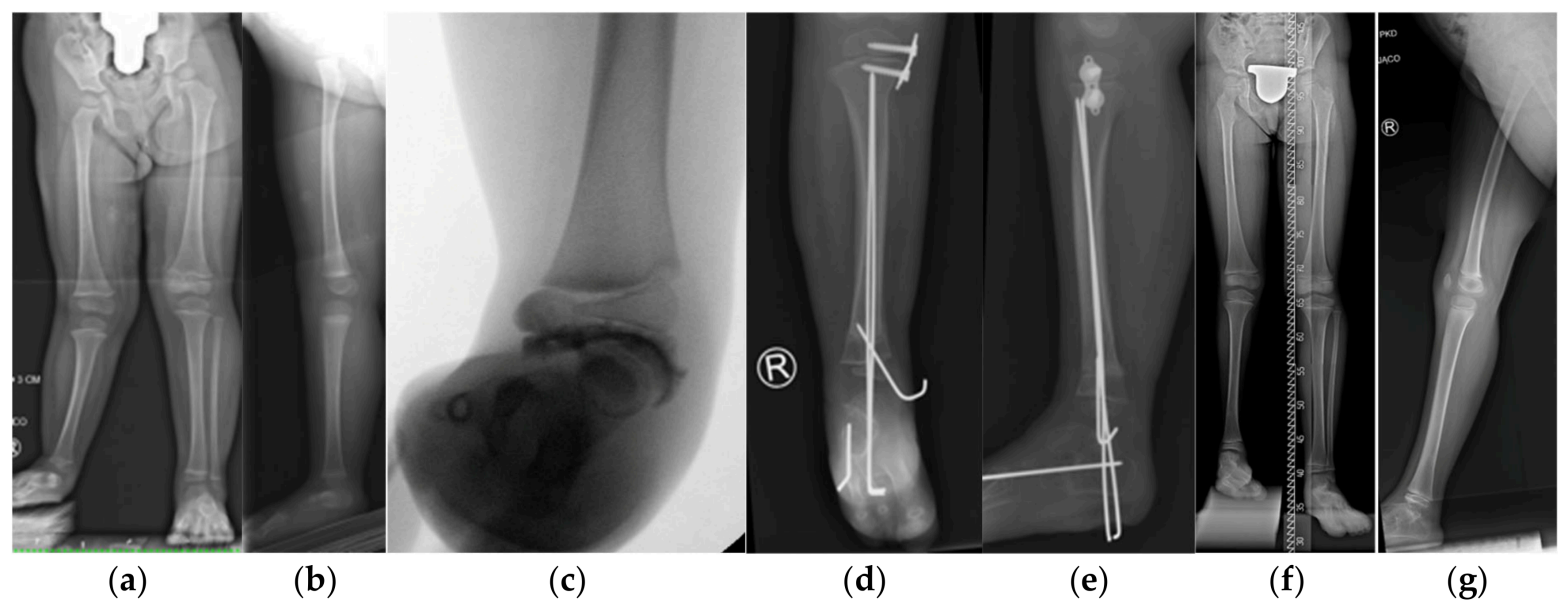
Radiographic evaluation of type 3C FH: (**a**,**b**) preoperative radiograph; (**c**) ankle arthrography; (**d**,**e**) radiograph obtained 6 weeks after SUPERankle procedure; (**f**,**g**) radiograph obtained 1 year postoperatively. R—right lower limb, NA STOJĄCO—standing position.

Fixation is typically achieved with Kirschner wires (maintained for 6 weeks), followed by casting for approximately 3 months. In older children, an external fixator may be preferred (Figure 17 and Figure 18).

#### 3.5.2. Limb Lengthening in FH

Lengthening is initiated after preparatory surgery, usually between ages 3 and 4. Typical LLD in FH ranges from 20–26 cm, requiring three staged lengthenings [30]:

- First lengthening: age 3–4 years (≈5 cm);
- Second lengthening: age 7–10 years (5–8 cm);
- Third lengthening: age 12–14 years (5–8 cm).

**Figure 19:**
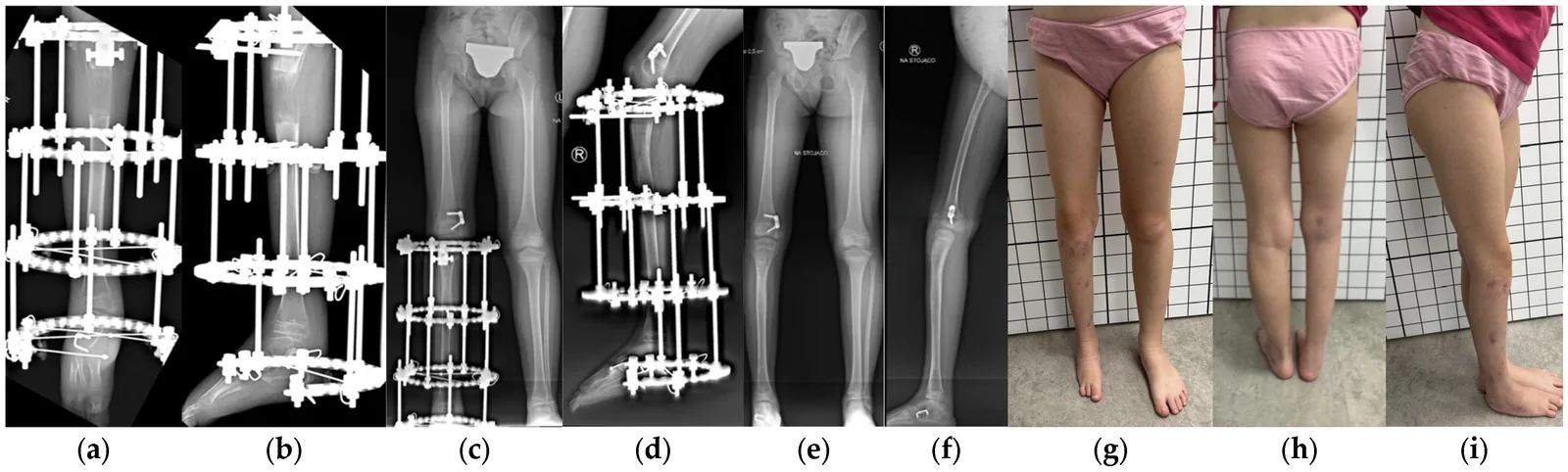
Limb lengthening in the same patient: (**a**,**b**) first lengthening using the Ilizarov method with foot fixation; (**c**,**d**) consolidation phase after 5 cm lengthening; (**e**,**f**) radiographic result; (**g**–**i**) clinical result. R—right lower limb, NA STOJĄCO—standing position.

During lengthening, spanning the external fixator across the ankle provides additional protection (Figure 19).

#### 3.5.3. Additional Procedures

In FH, growth modulation for knee valgus, ACL/PCL reconstruction (especially before femoral lengthening), correction of associated CFD when present, and ankle fusion for severe deformity or persistent subluxation may be required. Ultimately, treatment must be individualized, guided by functional goals and family expectations.

## 4. Tibial Hemimelia (TH)

**Figure 20:**
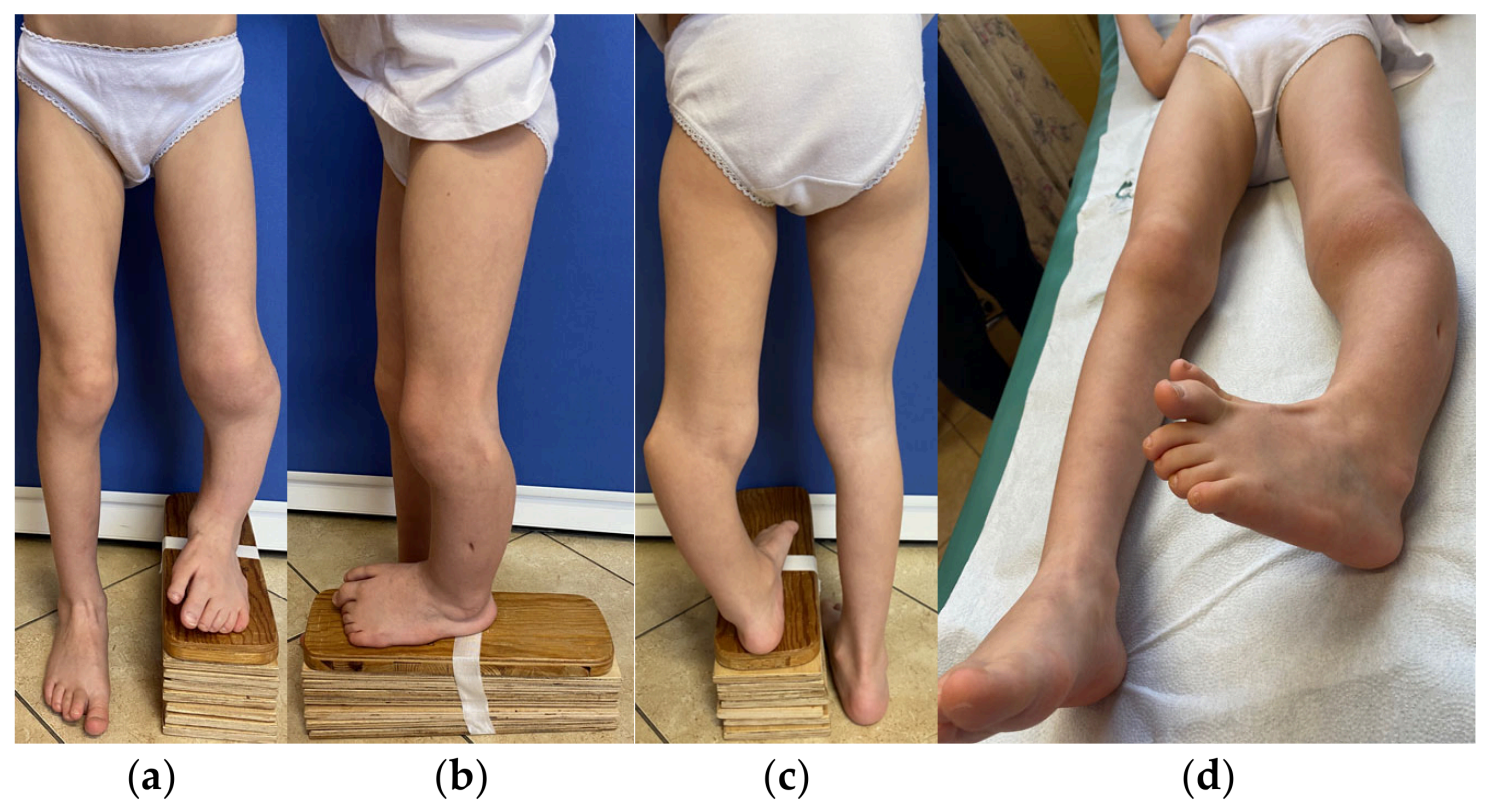
(**a**–**d**) Clinical view of TH type IIB with 8 cm shortening, knee varus, foot varus, accessory toe ray, and double talus.

Tibial hemimelia (TH) is an extremely rare congenital longitudinal deficiency of the lower limb, clinically presenting with partial or complete absence of the tibia, variable development of the tibial anlage, and a heterogeneous pattern of associated anomalies. Common features include knee flexion contracture, absence or hypoplasia of the patella and quadriceps mechanism, instability of both the knee and ankle joints, clubfoot deformity, polydactyly, duplicated great toe, and significant limb-length discrepancy (Figure 20) [37].

### 4.1. Diagnostic Imaging

**Figure 21:**
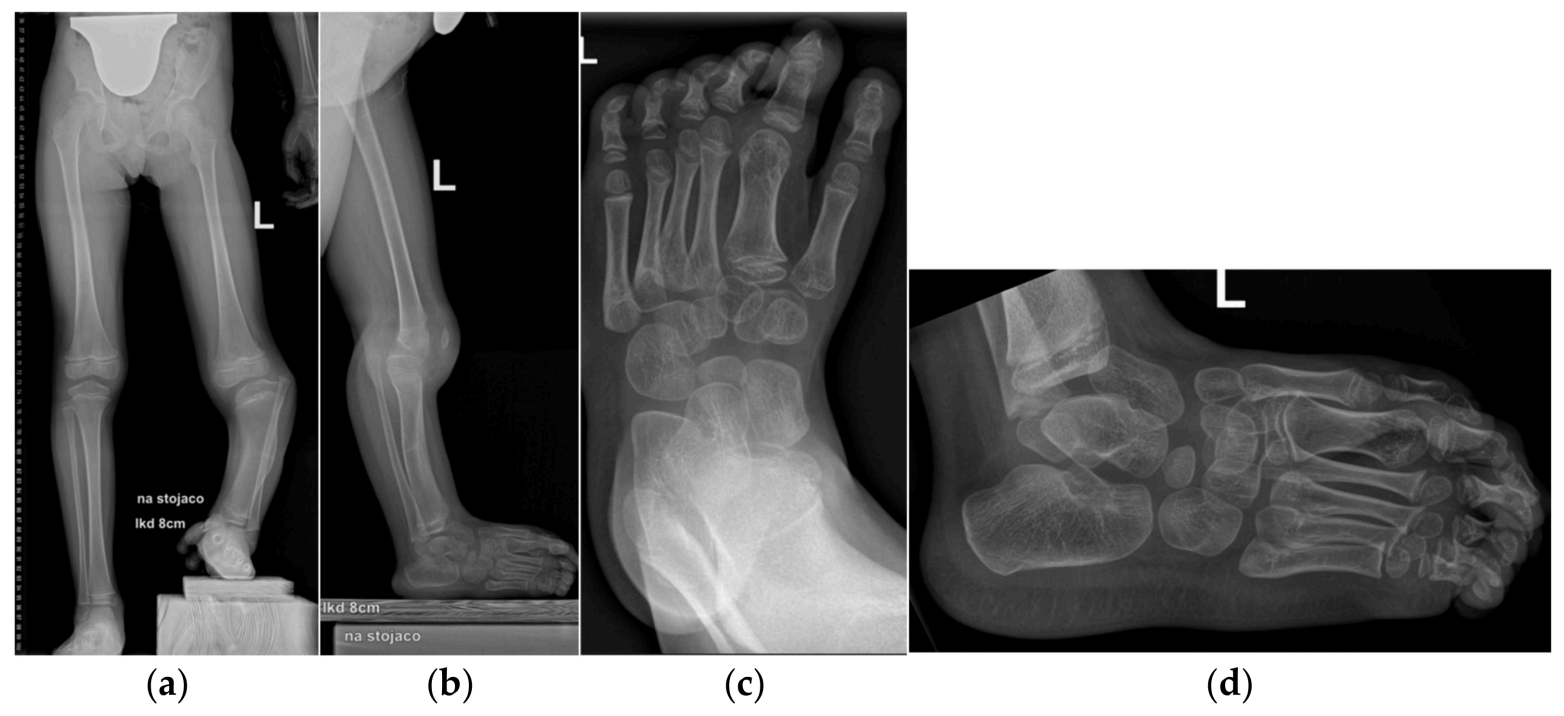
Radiographic views of type IIB TH: (**a**) knee varus with subluxation and 8 cm shortening; (**b**) fibular overlengthening; (**c**) varus foot deformity; (**d**) accessory toe ray with double talus. lkd—left lower limb, na stojąco—standing position, L—left.

Radiographic assessment in tibial hemimelia follows the same principles described for congenital femoral deficiency (CFD), including standard AP and lateral radiographs, standing full-length radiographs when feasible, and the selective use of MRI and CT for detailed evaluation and surgical planning. In tibial hemimelia, however, special consideration should be given to young children in whom the tibial anlage may be cartilaginous and non-ossified, rendering it invisible on radiographs. In these cases, MRI or ultrasonography is particularly valuable for visualizing the cartilaginous anlage, soft-tissue anatomy, and joint morphology (Figure 21) [37,38].

### 4.2. Classification Systems

Several classification systems have been proposed for tibial hemimelia, evolving from simple anatomical descriptions to function-based systems that guide reconstruction [3,37,39,40,41].

The Jones classification remains the most widely used because of its simplicity and radiographic basis. It classifies tibial hemimelia into four major types according to the presence or absence of the proximal and distal tibia. Its principal advantage is ease of use; however, it provides limited information regarding treatment planning [39].

The Kalamchi–Dawe classification expands on the Jones system by providing a more detailed anatomical description and recognizing rare variants such as tibial duplication. Although it improves anatomical characterization, it remains largely descriptive and offers little guidance for surgical decision-making [40].

The Weber classification is the most comprehensive anatomical system. It incorporates both ossified and cartilaginous structures, as well as associated foot abnormalities, enabling detailed assessment of deformity severity. However, its complexity makes it difficult to memorize and less practical for everyday clinical use. As a result, despite its detailed nature, it has not achieved widespread adoption in routine orthopedic practice [3].

The Paley classification represents a major shift toward reconstruction-oriented assessment. By emphasizing quadriceps function, knee stability, and limb reconstructability. It is useful systems for reconstructive planning [37].

More recently, the Hosny classification introduced a functional framework that combines tibial morphology with quadriceps integrity, knee stability, ankle stability, and growth potential. Patients are categorized according to both anatomical severity and functional prognosis. The Hosny classification represents the latest evolution in tibial hemimelia classification, integrating anatomical and functional factors to optimize reconstruction-guiding decisions. Its principal limitation is its recent introduction, meaning that widespread validation and clinical adoption are still evolving [41].

Paley classification updated in 2016 is currently preferred because it captures the full spectrum of associated pathology and provides direct guidance for reconstructive strategies [37,38]. Each type and subtype correspond to specific treatment protocols.

**Figure 22:**
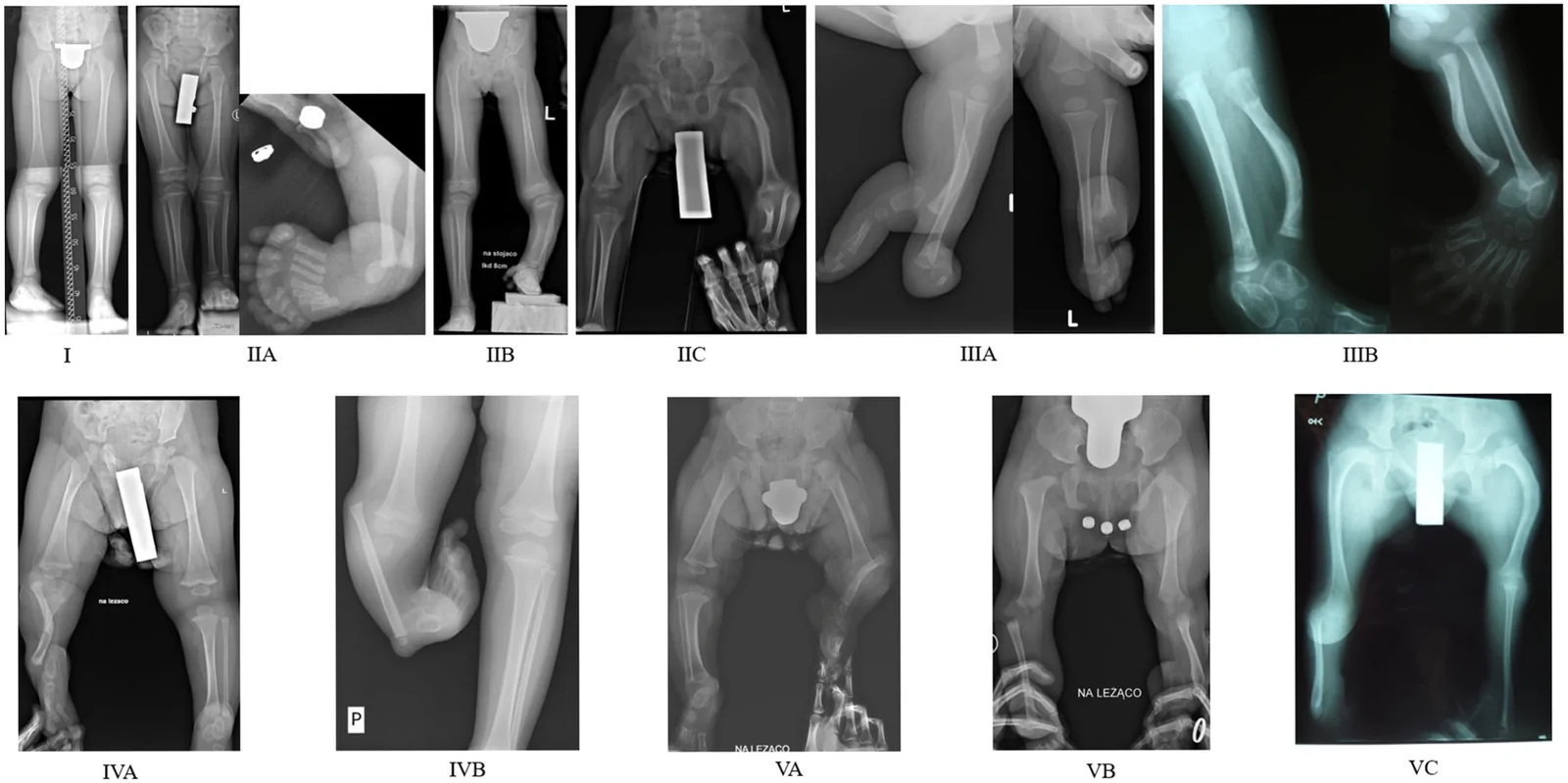
Types of TH according to the Paley classification (description provided in the text). lkd—left lower limb, na stojąco—standing position, NA LEŻĄCO—supine position, L—left.

The Paley classification (Figure 22) divides TH into the following types:

- Type I: Hypoplastic but continuous tibia with valgus deformity and relative proximal fibular overgrowth. Ankle plafond is normal;
- Type IIA: Proximal and distal tibial epiphyses are present, but the ankle is dysplastic; distal physis is separate from the proximal physis; tibial plafond is dysplastic; proximal fibula is overgrown;
- Type IIB: “Delta tibia” formed by a bracket epiphysis bridging the proximal and distal physis; malorientation of both ankle and knee; fibular overgrowth;
- Type IIC: Delayed ossification of a distal tibial anlage; distal tibial physis absent; ankle dysplastic; fibular overgrowth;
- Type IIIA: Proximal tibia intact; distal tibial plafond absent; medial and lateral malleoli present; tibia shows varus diaphyseal bowing; distal fibula and foot are internally rotated around the tibia; talus may lie between the tibia and fibula;
- Type IVA: Complete aplasia of the distal tibia beginning at the diaphysis; knee joint present; fibula is relatively overgrown;
- Type IVB: Proximal tibial epiphysis present but proximal physis absent; delayed epiphyseal ossification; fibular overgrowth;
- Type V: Complete absence of the tibia; VA: patella present; VB: patella absent but quadriceps and knee capsule present; VC: patella absents, quadriceps absent, knee capsule absent.

### 4.3. Treatment Strategy

Accurate classification is essential for determining treatment feasibility and designing a reconstructive plan. Key factors include presence and function of the extensor mechanism, detailed foot morphology and stability, limb-length measurement, and prediction of future discrepancy and reconstructability of the knee and ankle joints. Because TH presents with extreme variability, not all types are reconstructable. In complete tibial aplasia (e.g., Paley Type VC), amputation remains the most common and predictable treatment option. Overall, treatment must be individualized and based on family expectations, functional goals, and the reconstructive possibilities [38,42,43].

#### Treatment by Type of TH

Type I. The primary deformities are tibial shortening and proximal valgus. Management includes temporary medial proximal tibial hemiepiphysiodesis to correct valgus, proximal fibular epiphysiodesis (using screw) if the fibula is overgrown, and correction of limb-length discrepancy through tibial lengthening or contralateral epiphysiodesis, depending on the magnitude of shortening.

Type IIA. The foot is fixed in equinovarus and internally rotated. Treatment consists of gradual correction with an external fixator to derotate and realign the foot, followed by tibial osteotomy with lengthening until the tibia and fibula reach equal length.

Type IIB. Treatment includes resection of the bracket epiphysis, acute tibial osteotomy, fibular-shortening osteotomy to correct varus, and staged lengthening using an external fixator spanning the femur and upper tibia.

Type IIC. This type is characterized by delayed ossification of the distal tibial anlage. Treatment follows the Type IIA protocol, with the addition of bone morphogenetic protein (BMP) insertion into the tibial anlage during lengthening to stimulate ossification.

Type IIIA. This type is characterized by distal tibiofibular diastasis and internal rotation of the foot around the tibia. Treatment includes gradual distraction using an external fixator, combined with derotation of the foot and fibula, correction of the equinovarus deformity to plantigrade by positioning the talus beneath the tibia, and final open surgery to reshape the articular surfaces of the talus and distal tibia and close the tibiofibular diastasis using syndesmotic sutures.

Type IIIB. Treatment the same as type IIIA, with the addition of closure of the skin cleft between the tibia and fibula.

Type IVA. The knee joint and proximal tibial physis are intact and normally ossified. Treatment includes fibular osteotomy and proximal transfer of the distal fibula, fixation with intramedullary K-wires, correction of severe equinovarus deformity either acutely by soft-tissue release, or gradually through distraction and positioning of the talus beneath the fibula, and creation of a talus–distal fibula fusion while preserving the distal fibular physis.

**Figure 23:**
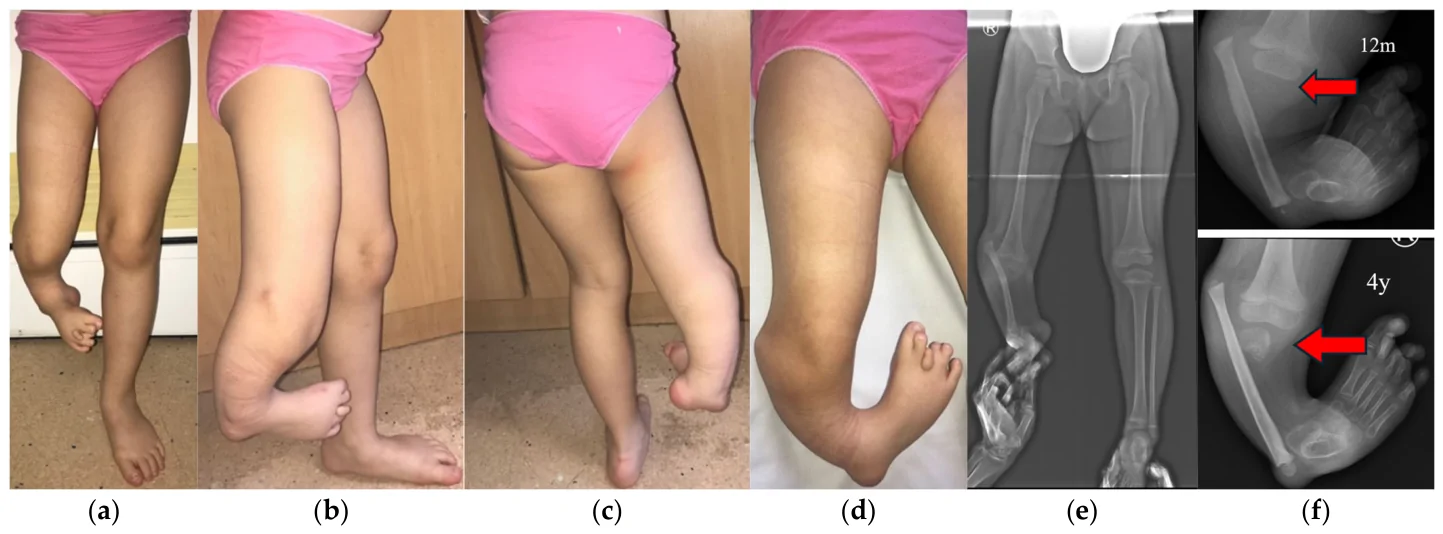
Clinical and radiographic views of right-sided type IVB TH: (**a**–**d**). Severe shortening, knee instability (**e**), equinovarus foot deformity, polydactyly (**f**). Radiographic view at 12 months and at 4 years of age presenting delayed epiphyseal ossification, and fibular over-growth. Red arrow is pointing the nucleus of ossification at the age of 12 months and 4 years. R—right.

**Figure 24:**
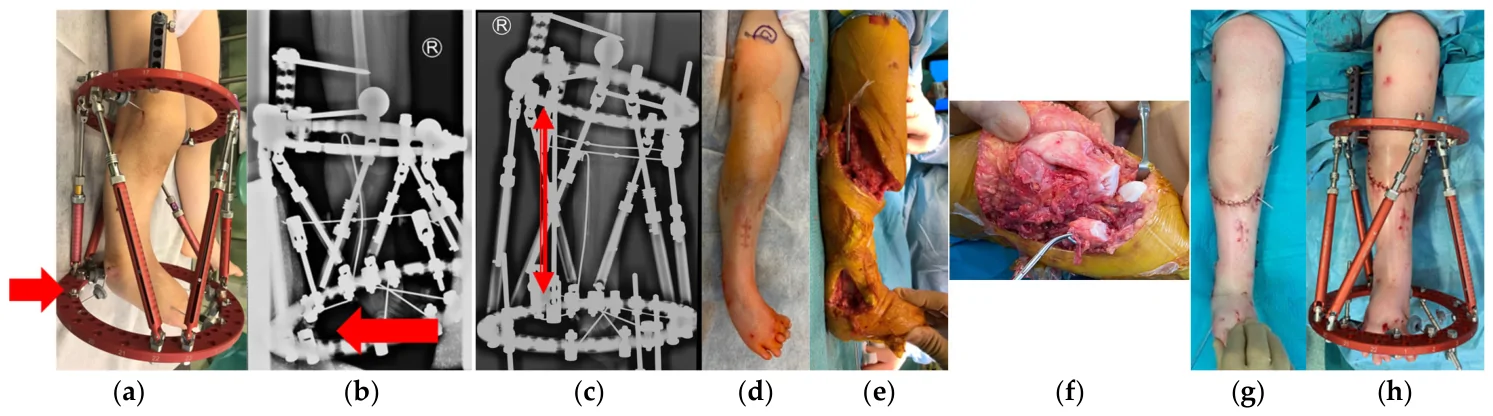
Staged reconstruction procedure in TH: (**a**) 1st stage—application of an external frame to the femur and foot with gradual axial distraction of the fibula for 5.5 cm; (**b**,**c**) 2nd stage—detachment of the fibular axial wire from the distal ring, fixation to the proximal ring using threaded rods and nuts, and gradual correction of varus deformity; (**d**–**f**) 3rd stage—docking and fixation of the fibula to the proximal tibial epiphysis and distal fixation to the talus; (**g**,**h**) reapplication of the external frame. (**a**,**b**) red arrows pointing the fixation of the fibula to the foot ring with the K-wires; (**c**) red arrow pointing the fixation of the fibular K-wire to the threaded rod, which is fixed to the proximal ring. R—right.

**Figure 25:**
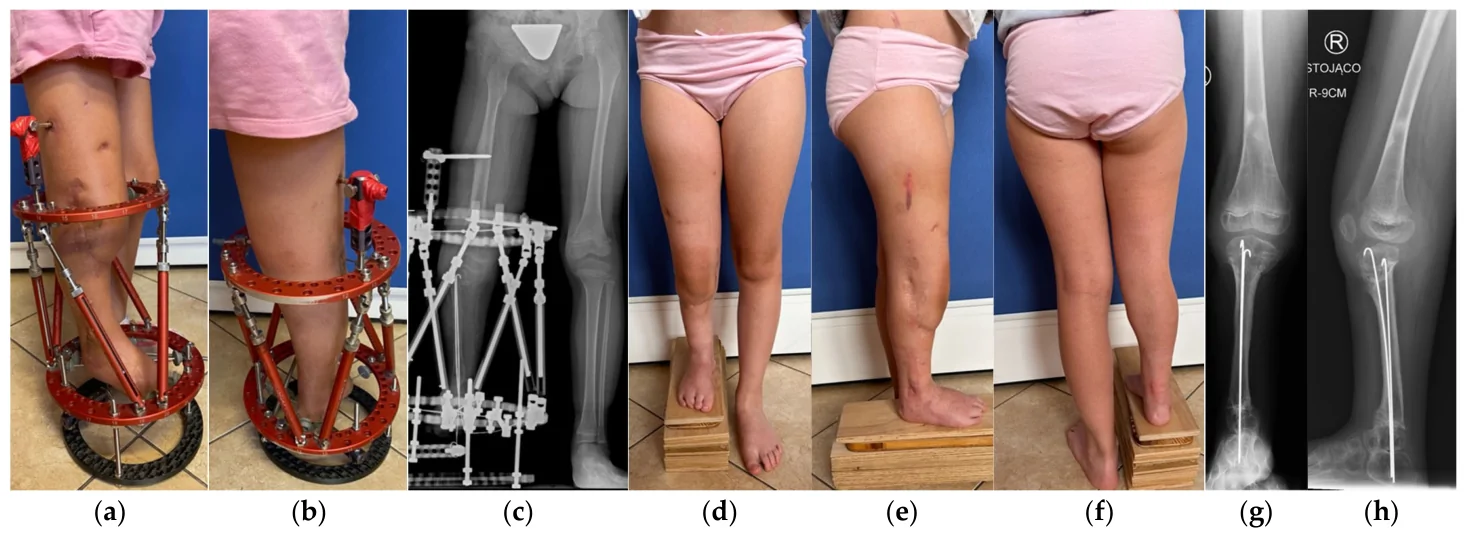
Foot correction and stabilization using an external frame: (**a**,**b**) clinical and (**c**) radiographic view; (**d**–**f**) clinical and (**g**,**h**) radiographic result. PKD—right lower limb, NA STOJĄCO—standing position, R—right.

**Figure 26:**
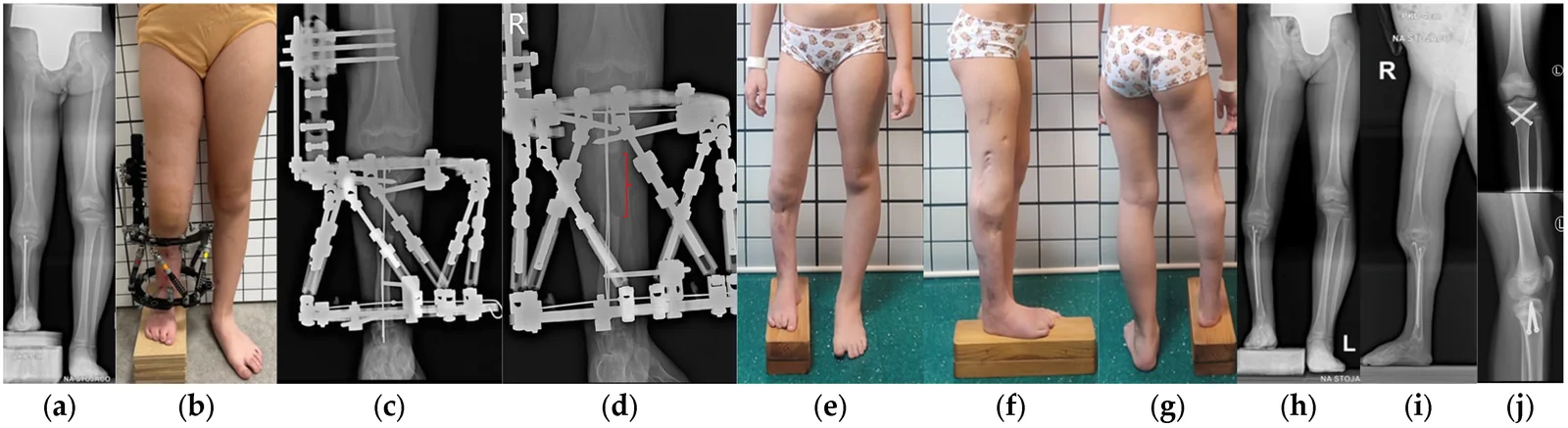
Fibular lengthening procedure: (**a**) preoperative appearance; (**b**) clinical view during the first 6 cm fibular lengthening using an external fixator; (**c**,**d**) radiographic view during lengthening; (**e**–**g**) clinical result; (**h**,**i**) radiographic result; (**j**) growth arrest of the contralateral tibia. NA STOJĄCO—standing position, L—left, R—right, red curly brace—bone regenerate.

Type IVB. The proximal tibial anlage is unossified, although the extensor mechanism is present, and the fibula is highly migrated. Treatment follows the same approach as for Type IVA but begins with external fixation to distract the fibula and stabilize the knee. The fibula is then osteotomized and transferred to the tibial anlage, and BMPs are inserted to promote ossification of the anlage (Figure 23, Figure 24, Figure 25 and Figure 26).

Type V. Complete absence of the tibia; the extensor mechanism may or may not be present.

When the extensor mechanism is absent two options of management are available: amputation, or centralization of the fibula to create a femoro-fibulo-calcaneal arthrodesis. Although the latter causes the knee and foot to be stiff, this sort of treatment is acceptable by the patient. If the extensor mechanism is present reconstruction is possible using modified Weber patellar arthroplasty (Paley modification). In the Paley modification the fibula is gradually distracted to realign and lengthen the leg, the patella is transferred to the distal femur and fused to the fibular head, creating a stable neo-knee joint surface. This approach preserves knee function and may reduce the need for amputation.

## 5. Discussion

Recent advances in reconstructive surgery, deformity correction, and limb-lengthening techniques have significantly improved outcomes in congenital lower limb deficiencies. In congenital femoral deficiency, staged reconstruction can achieve substantial limb-length equalization and satisfactory joint function, although residual proximal femoral deformity, abnormal femoral version, hip instability, and complications related to repeated lengthening remain important concerns [21]. Fibular hemimelia generally demonstrates the most predictable reconstructive outcomes, with contemporary protocols incorporating ankle stabilization, deformity correction, guided growth, ligament reconstruction, and limb lengthening enabling preservation of a stable plantigrade foot, restoration of limb alignment, and high levels of patient satisfaction [30,31,44,45]. Recent studies have reported successful outcomes even in severe forms of fibular hemimelia, emphasizing the importance of ankle stability and early intervention in optimizing long-term function [31,45]. In tibial hemimelia, successful limb salvage depends largely on preservation of quadriceps function, knee stability, and foot reconstructability. Modern reconstruction techniques and classification-guided treatment strategies have expanded the indications for limb preservation; however, treatment remains complex and is frequently associated with recurrent deformity, joint stiffness, and the need for multiple secondary procedures [38,42,46]. Despite these challenges, recent studies have demonstrated favorable functional outcomes, satisfactory quality of life, and durable long-term results following reconstruction in carefully selected patients [42,46]. Increasingly, treatment success is evaluated using patient-reported outcome measures (PROMs), and contemporary evidence suggests that radiographic correction alone does not necessarily correlate with patient satisfaction. Rather, mobility, independence, participation in daily activities, psychosocial well-being, treatment burden, and overall health-related quality of life are increasingly recognized as the principal determinants of successful long-term outcomes across all forms of congenital limb deficiency [42,46].

When discussing treatment strategies and outcomes for complex congenital deformities, it is also important to address the role of fully implantable lengthening nails. The introduction of motorized intramedullary lengthening nails has transformed limb reconstruction by enabling gradual bone lengthening without prolonged external fixation. Studies have demonstrated high accuracy and precision of lengthening, reliable bone healing, successful correction of limb-length discrepancy, and favorable functional outcomes [47,48,49]. Compared with external fixation, implantable nails substantially reduce pin-site infections, soft-tissue complications, pain, and treatment-related discomfort while facilitating rehabilitation and preserving joint mobility [50,51]. Beyond radiographic and functional outcomes, recent evidence highlights significant improvements in PROMs and health-related quality of life. Patients treated with motorized intramedullary nails report lower treatment burden, greater comfort, improved self-image, better cosmetic acceptance, and earlier return to school, work, sports, and recreational activities compared with traditional external fixation methods [50,51]. Reduced pain, improved mobility, and the absence of an external frame also contribute to better psychological well-being, social participation, and overall treatment satisfaction. Consequently, many patients report high levels of quality of life during and after treatment, with favorable long-term functional and psychosocial outcomes.

However, implantable lengthening nails have limitations, including the need for adequate bone size and a minimum femoral intramedullary canal diameter of approximately 11 mm, which may restrict their use in younger children and patients with severe congenital deformities. Although complications such as delayed consolidation, insufficient regenerate formation, joint contractures, and occasional mechanical failure have been reported, the overall complication profile is generally lower than that associated with external fixation techniques [48,50]. Therefore, motorized intramedullary nails have become an important component of contemporary reconstruction strategies for congenital femoral deficiency and selected cases of fibular hemimelia, providing reliable lengthening with improved patient comfort, quality of life, and patient satisfaction.

The treatment of complex congenital lower limb deficiencies involves not only advanced and technically demanding reconstructive procedures but also fundamental techniques, such as guided growth. Guided growth (temporary or permanent epiphysiodesis) is a well-established treatment for angular deformities and moderate limb-length discrepancy (LLD) in skeletally immature patients and is typically indicated for predicted discrepancies of 2–6 cm at skeletal maturity [52]. Owing to its minimally invasive nature, guided growth is associated with lower morbidity, shorter recovery, and fewer complications than reconstructive or lengthening procedures. Common techniques include permanent epiphysiodesis and temporary growth modulation using transphyseal screws, staples, or tension-band plates, with recent evidence favoring percutaneous epiphysiodesis screws because of their high success rates and low complication profile [52].

In children with congenital lower limb deficiencies, guided growth is particularly useful for correcting progressive coronal plane deformities and managing residual LLD during growth. Recent studies have demonstrated the effectiveness of hemiepiphysiodesis in congenital femoral deficiency and postaxial lower limb deficiencies, allowing simultaneous correction of angular deformity and optimization of limb alignment while reducing the need for additional surgical procedures [53,54]. Guided growth may also reduce the need for subsequent lengthening procedures when a moderate discrepancy persists after reconstruction. However, outcomes depend on accurate growth prediction and sufficient remaining growth, and correction may be less predictable in congenital conditions because of abnormal physeal morphology and altered growth patterns. Potential complications include rebound deformity, recurrence, undercorrection, and overcorrection, necessitating close follow-up until skeletal maturity [54]. Because guided growth does not address substantial shortening, joint instability, or complex foot and ankle deformities, it should be regarded as part of a comprehensive treatment strategy rather than a standalone solution. Contemporary evidence supports guided growth as a safe and effective adjunct within modern limb reconstruction programs, particularly when integrated with deformity correction and limb-lengthening procedures [53].

One of the most debated issues in the management of congenital lower limb deficiencies remains reconstruction versus amputation. Advances in deformity correction, guided growth, limb lengthening, ligament reconstruction, ankle stabilization, and implantable lengthening technologies have expanded the indications for limb salvage, allowing many patients to achieve a plantigrade foot, improved alignment, joint stability, and satisfactory functional outcomes [22,23,30,44].

Historically, amputation was commonly recommended for severe fibular and tibial hemimelia because reconstructive options were limited. Contemporary reconstruction can preserve the limb and improve function but often requires multiple operations, prolonged rehabilitation, and long-term follow-up, with ongoing risks of recurrent deformity, joint instability, residual limb-length discrepancy, and lengthening-related complications [22,30]. Recent studies have demonstrated favorable outcomes following complex reconstructive procedures, including simultaneous ACL reconstruction and limb lengthening, as well as correction of severe ankle malalignment in advanced fibular hemimelia, further supporting the expanding role of limb salvage in appropriately selected patients [31,44]. Conversely, modern prosthetic management following Syme or Boyd amputation can provide reliable mobility, efficient gait, and early functional independence while avoiding years of repeated surgical interventions [34]. Nevertheless, amputation entails lifelong prosthetic dependence, socket-related complications, and periodic prosthetic replacement.

Current evidence does not demonstrate clear superiority of either strategy. Comparative studies have shown that both reconstruction and amputation can achieve satisfactory physical function, psychosocial well-being, quality of life, and patient-reported outcomes when appropriately indicated [34]. Contemporary reviews further emphasize that advances in reconstructive techniques have substantially improved long-term outcomes compared with historical treatment methods, particularly when a stable plantigrade foot and good joint alignment can be achieved [23,30]. Therefore, treatment decisions should be individualized and based on deformity severity, reconstructive feasibility, expected treatment burden, patient and family preferences, and local expertise. Shared decision-making within a multidisciplinary team remains essential to optimize long-term outcomes [30,34].

Although limb reconstruction and lengthening have significantly improved outcomes in children with congenital lower limb deficiencies, treatment remains associated with a substantial risk of complications and often requires repeated procedures during growth [55,56]. Common complications include recurrent deformity, residual limb-length discrepancy, delayed or premature regenerate consolidation, axial deviation, nonunion, fractures after frame removal, joint instability, pin-tract infection, soft-tissue contracture, joint stiffness, and neurovascular complications [55,56]. Recent studies have also highlighted hardware-related complications, including locking screw migration, implant breakage, distraction mechanism failure, and loss of fixation requiring revision surgery, particularly with motorized intramedullary lengthening systems [57]. Despite these challenges, long-term outcomes are generally favorable, with most patients achieving satisfactory alignment, improved limb-length equality, independent ambulation, and good functional outcomes. However, assessment of treatment success should extend beyond radiographic correction to include gait, joint function, PROMs, and health-related quality of life [57,58].

Another issue is the psychosocial burden of limb reconstruction. Limb reconstruction for congenital lower limb deficiencies is often a prolonged process requiring multiple surgeries, rehabilitation, and long-term follow-up. Beyond achieving deformity correction and limb-length equalization, treatment may impose significant psychological, social, and emotional burdens on both patients and families. Children can experience anxiety, pain, body-image concerns, reduced participation in school and recreational activities, and challenges related to external fixation and repeated hospitalizations, while parents often face considerable stress associated with long-term care and uncertainty regarding outcomes [59]. Importantly, the psychological impact of both the deformity and its prolonged treatment may be long-lasting, persisting into adolescence and adulthood, and may continue to affect self-image, social functioning, and quality of life. Despite these challenges, most patients report satisfactory long-term adaptation, quality of life, and psychosocial well-being when supported by family involvement, multidisciplinary care, and appropriate psychological support [58]. Consequently, treatment success should be evaluated not only by radiographic and functional outcomes but also by PROMs, psychological well-being, social participation, and health-related quality of life [59,60].

Another important issue is the critical evaluation of the available literature, with particular emphasis on the level of evidence and remaining knowledge gaps. Despite substantial advances in limb reconstruction, the current evidence base for congenital lower limb deficiencies remains limited. Most published studies are retrospective case series or cohort studies from specialized centers, corresponding primarily to Level III–IV evidence, with relatively small patient populations due to the rarity of these conditions [30,61]. Comparison between studies is further constrained by heterogeneity in patient selection, deformity severity, classification systems, surgical techniques, and rehabilitation protocols [30].

Although modern reconstructive methods, including guided growth, computer-assisted hexapod external fixation, and motorized intramedullary lengthening nails, have demonstrated encouraging outcomes, most investigations emphasize radiographic correction, limb-length equalization, and complication profiles rather than long-term functional outcomes, PROMs, psychosocial well-being, and health-related quality of life [61,62]. Recent studies have also reported favorable outcomes following complex lengthening strategies for severe fibular hemimelia; however, these studies remain retrospective, involve relatively small cohorts, and provide only short- to mid-term follow-up, limiting the generalizability of their findings [62,63].

Furthermore, comparative evidence evaluating limb reconstruction versus amputation and outcomes extending into adulthood remains scarce. Current reviews continue to emphasize the lack of prospective comparative studies and the ongoing reliance on institutional treatment algorithms and expert consensus for clinical decision-making [30].

Consequently, many treatment recommendations are still based on expert opinion and specialized-center experience rather than high-level evidence. Future research should prioritize prospective multicenter collaborations, standardized outcome measures, routine PROM assessment, and long-term follow-up into adulthood to establish more robust evidence-based treatment guidelines [30,61].

## 6. Conclusions

Congenital lower limb deficiencies—such as congenital femoral deficiency, fibular hemimelia, and tibial hemimelia—are complex pediatric orthopedic conditions characterized by limb-length discrepancy, joint instability, limb deformity, and functional impairment. Modern management prioritizes staged, carefully sequenced reconstruction that emphasizes joint stability and soft-tissue balance before limb lengthening to reduce complications. Accurate clinical assessment, advanced imaging, and use of the Paley classification systems are crucial to treatment planning. Specialized procedures (SUPERhip, SUPERknee, and SUPERankle) address joint deformities and instability, followed by staged limb lengthening. While reconstruction can restore function and limb length in many cases, recurrence, instability, and complications remain significant challenges, and in severe or non-reconstructable cases, amputation may provide the most reliable functional outcome.

The learning curve is a critical factor in the treatment of congenital lower limb deficiencies, as these rare conditions require complex reconstructive techniques, and outcomes are strongly influenced by surgical experience and management in specialized centers.

## Data Availability

No new data were created or analyzed in this study. Data sharing is not applicable to this article.

## Abbreviations

The following abbreviations are used in this manuscript:

ACL	Anterior Cruciate Ligament
AI	Acetabular Index
AP	Anteroposterior
BMP	Bone Morphogenetic Protein
CEA	Center-Edge Angle
CFD	Congenital Femoral Deficiency
CT	Computed Tomography
DSM	Discrepancy at Skeletal Maturity
FH	Fibular Hemimelia
LLD	Limb Length Discrepancy
MRI	Magnetic Resonance Imaging
PCL	Posterior Cruciate Ligament
SUPER	Systematic Utilitarian Procedure for Extremity Reconstruction
TH	Tibial Hemimelia
